# Supplementary material for: Safe CNV removal is crucial for successful hESC-RPE transplantation in wet age-related macular degeneration
Source: Stem Cell Reports. 2025 Feb 27;20(3):102424. doi: 10.1016/j.stemcr.2025.102424 (PMC11960522; doi:10.1016/j.stemcr.2025.102424)
Supplement: Document S1. Figures S1–S6, Tables S1 and S2, and supplemental methods [file mmc1.pdf]

Stem Cell Reports, Volume *20*

Supplemental Information

Safe CNV removal is crucial for successful hESC-RPE transplantation in wet age-related macular degeneration

Ying Xue Lv, Qi You Li, Ping Duan, Min Fang Zhang, Bo Liu, Shi Ying Li, Tong Tao Zhao, Hao Wang, Yong Liu, and Zheng Qin Yin

Supplemental Information

Supplemental Figures

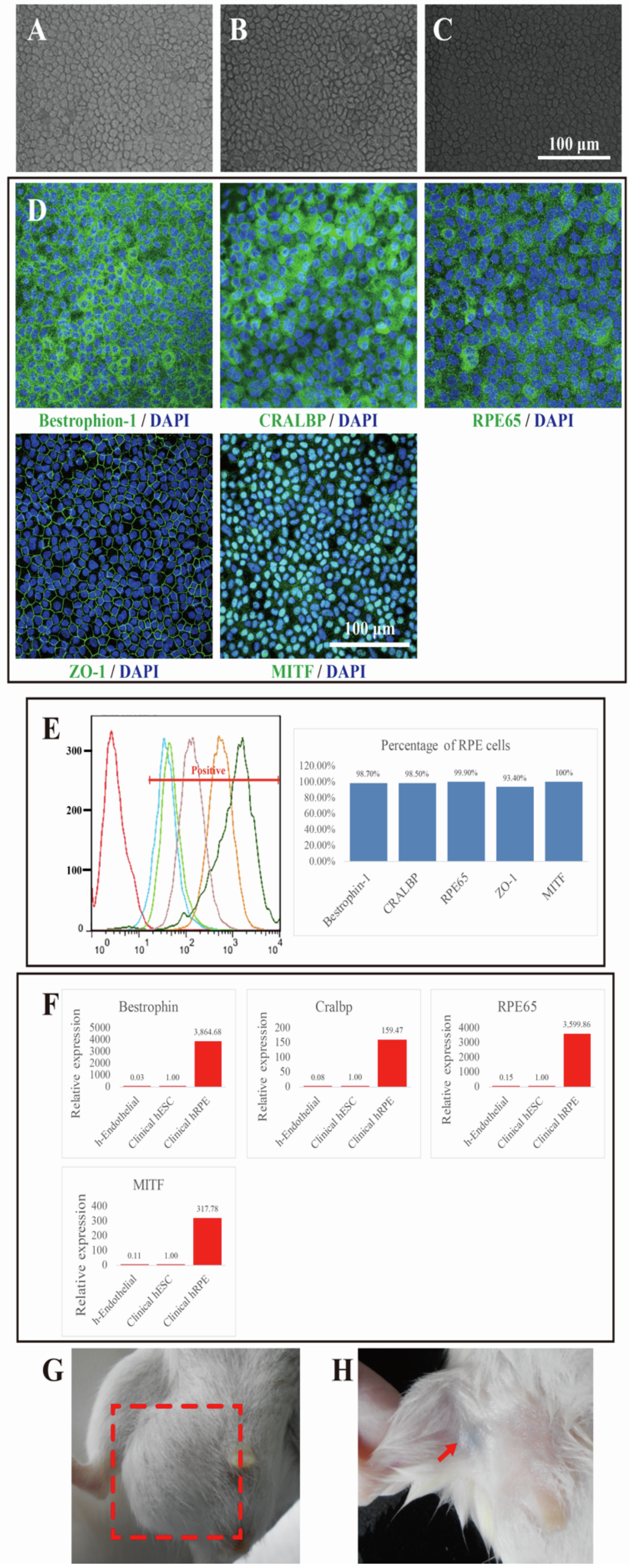

Figure S1. hESC-RPE cell identification of transplantation for patients. (A-C) Brightfield microphotography of induced hESC-RPE at passage 3 shows that the confluent cobblestone monolayer with pigment accumulation (A-patient 1, B-patient 2, C-patient 3). (D) Immunofluorescence staining assay. The tight junction complex protein (ZO-1), early neural and eye field markers (MITF), RPE-specific markers (Bestrophin-1, CRALBP, RPE65) (green) with corresponding DAPI (blue). (E) Flow cytometric analysis. (F) Quantitative PCR shows up-regulation of RPE cell-specific genes, including Bestrophin-1, CRALBP, RPE65 and MITF. (G-H) Tumorigenicity testing in SCID mice. Three months after subcutaneously injection, teratomas developed in mouse with Q-CTS-hESC-2 cells (G, red dotted box), and no hyperproliferation or abnormal growth in mouse with CTS-hESC2-RPE cells (H, red arrow).

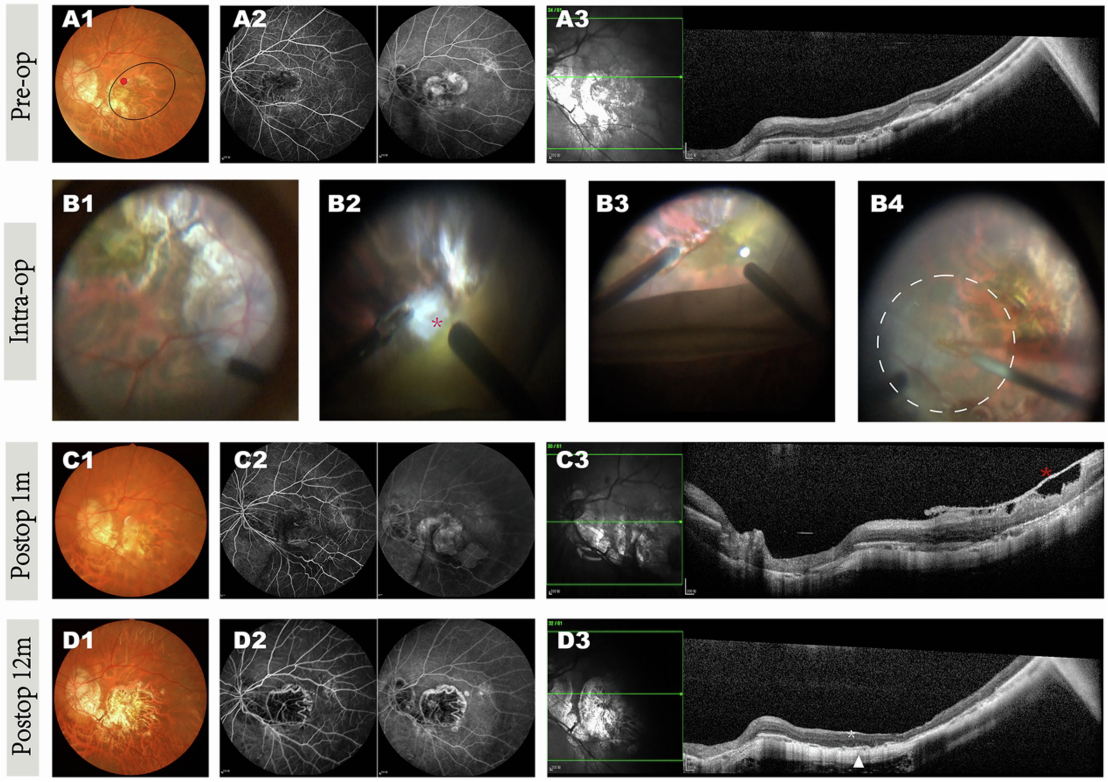

Figure S2. Retinal morphological changes in the operated eye of patient 2. Retinal examination before and after choroidal neovascularization (CNV) removal coupled with hESC-derived RPE suspension transplantation, at pre-operative (A1-A3), intra-operative (B1-B4), post-operative 1-month (C1-C3), and post-operative 12 months (D1-D3) stages. Pre-operative (A1-A3): (A1) Fundus photography showing the fibrotic membrane in the macular area. The injection site was marked by red dots and the formed subretinal bleb was indicated by a black circle. (A2) FFA images highlighting the typical shape of CNV. (A3) SD-OCT scan shows a hyperreflective mass broken through the RPE layer. The signal beneath the CNV was hypo-transmission. Intra-operative (B1-B4): (B1) Intraoperative image before incising the peripheral temporal quadrant retina. (B2) After incising the retina, the fiber membrane (red asterisk) is visible and easy to peel off without choroidal vessels damage. (B3) No fundus hemorrhage was observed after complete removal of the CNV membrane. (B4) A subretinal

bleb was formed after subretinal injection of transplanted cells, marked by a dashed circle. Post-operative 1-month (C1-C3): (C1) Fundus photo showing no subretinal pigmentation, hemorrhage or recurrent CNV, but macular geographic atrophy developed. (C2) The FFA shows no fluorescence leakage that suggestive of recurrent CNV network. (C3) SD-OCT image demonstrates no residual CNV lesion but development of ERM (asterisk) and retinal edema. Post-operative 6 months (D1-D3): (D1) FFA images reveal no subretinal pigmentation, hemorrhage or recurrent CNV, but macular geographic atrophy developed. (D2) The FFA shows no fluorescence leakage. (D3) SD-OCT image demonstrates a hyperreflective layer-like structure (arrowheads) over the bare Bruch’s membrane, with the relatively good restoration of ONL (white asterisk).

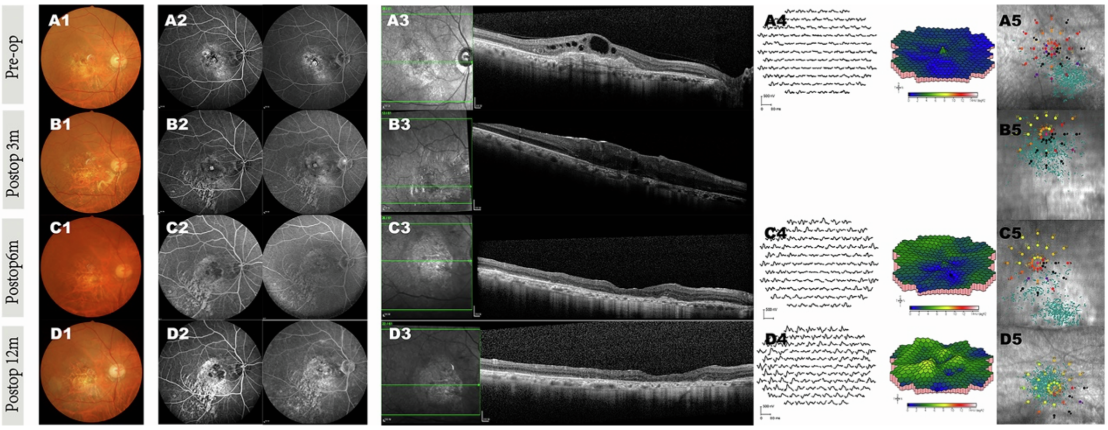

Figure S3. Retinal morphological and functional changes in the operated eye of patient 3. Fundus photography (1), FFA images (2), SD-OCT scan (3), mf-ERG (4) and microperimetry (5) at pre-operative (A), post-operative 3-month (B), post-operative 6-month (C), and post-operative 12 months (D) stages. Preoperatively, CNV lesion with cystoid macular edema were seen and FFA images indicated the leakage of the lesion. ERM occurred at 3 months and caused corresponding macular edema. After peeling the membrane, the retinal edema and the late fluorescence leakage subsided. No residual or recurrent CNV lesion was observed. Retinal function remained stable throughout the follow-up.

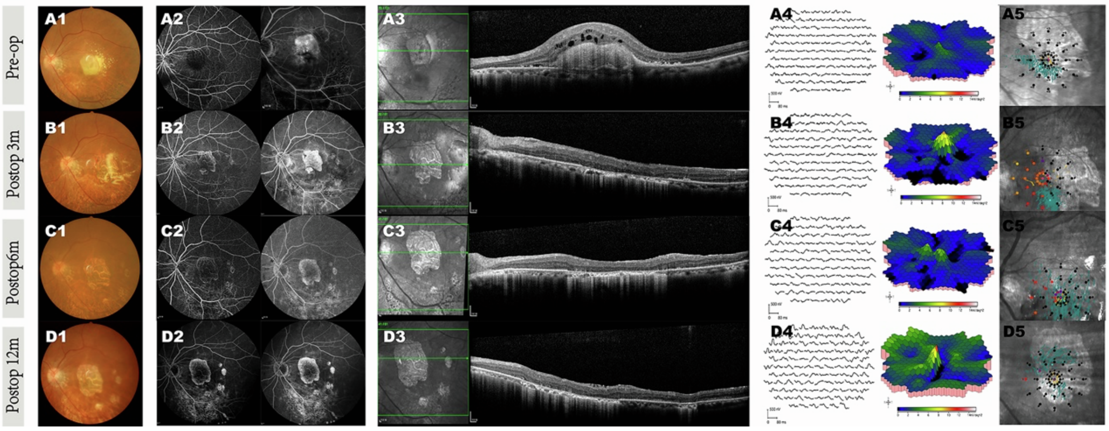

Figure S4. Retinal morphological and functional changes in the operated eye of patient 4. Fundus photography (1), FFA images (2), SD-OCT scan (3), mf-ERG (4) and microperimetry (5) at pre-operative (A), post-operative 3-month (B), post-operative 6-month (C), and post-operative 12 months (D) stages. Preoperatively, typical CNV lesion with retinal edema and late fluorescence leakage were presented. The retinal structure recovered with no recurrent edema or leakage postoperatively. No residual or recurrent CNV lesion was observed. Retinal function remained stable until the last visit.

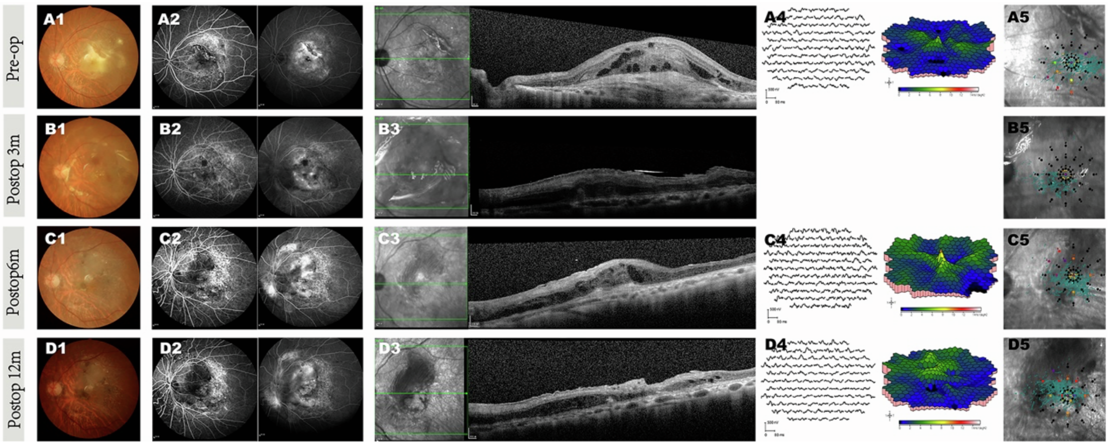

Figure S5. Retinal morphological and functional changes in the operated eye of patient 9. Fundus photography (1), FFA images (2), SD-OCT scan (3), mf-ERG (4) and microperimetry (5) at pre-operative (A), post-operative 3-month (B), post-operative 6-month (C), and post-operative 12 months (D) stages. Preoperatively, the fibrotic membrane and elevated retina were presented in the macular area. During surgery, hemorrhage occurred when removing the CNV. The retina edema persisted after ERM removal at 3 months postoperatively. After subsequent intravitreal TA injections, the retina became flatten and the fluorescence leakage subsided. No residual or recurrent CNV lesion were observed. Retina function remained stable throughout the follow-up.

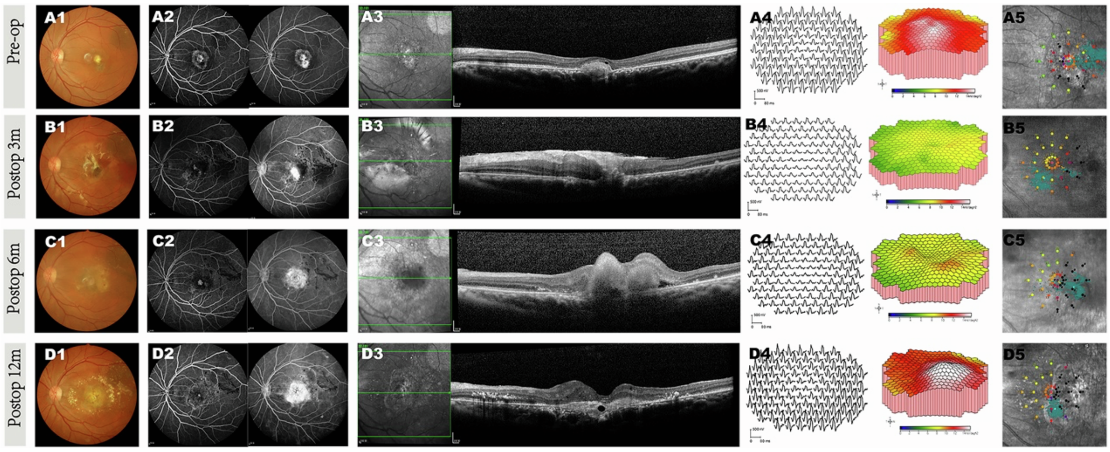

Figure S6. Retinal morphological and functional changes in the operated eye of patient 10. Fundus photography (1), FFA images (2), SD-OCT scan (3), mf-ERG (4) and microperimetry (5) at pre-operative (A), post-operative 3-month (B), post-operative 6-month (C), and post-operative 12 months (D) stages. Preoperatively, the fibrotic neovascular membrane under the RPE layer was visible, and FFA images showed the typical CNV shapes without obvious late fluorescence leakage. Spontaneous subretinal hemorrhage and increased fluorescence leakage in the late stage were observed. ERM peeling did not improve these presentations. However, after three consecutive anti-VEGF treatments, the retina became flattened, and the edema significantly subsided. The residual and recurrent CNV lesions were observed by OCTA. Retina function remained stable throughout the follow-up.

Supplemental Tables

Table S1. Q-CTS-hESC-2-RPE biosafety testing.

Test	Specification	Clinical hESC-RPE Cell
Fungus	Negative	Negative
Bacterium	Negative	Negative
Mycoplasma	Negative	Negative
Hepatitis B Virus (HBV)	Negative	Negative
Human Immunodeficiency Virus (HIV)	Negative	Negative
Hepatitis C Virus (HCV)	Negative	Negative
Treponema Pallidum (TP)	Negative	Negative
Endotoxin	<0.50EU/mL	<0.50EU/mL

Table S2. The sequences of the primers used to amplify hRPE cell specific genes.

Gene name	Forward primers	Reverse primers	Product size(bp)
MITF (NM_198159)	TTCACGAGCGTCCTGTATGCAGAT	AGTTTCCCGAGACAGGCAACGTAT	149
BESTROPHIN (NM_004183)	TATGACTCCGGCAGAACACAAGCA	TGTTCATCTCGTTCAGCAGGCTCT	161
RPE-65	TGCTTACGTACGGGCAATGACTGA	AGTTGGTCTCTGTGCAAGCGTAGT	191
CRALBP	TTCAAGGGCTTTACCATGCAGCAG	AGTACCATGGCTGGTGGATGAAGT	130
GAPDH (human)	agaaggctggggctcatttg	aggggccatccacagtcttc	

Supplemental Methods

hESC-RPE Cell Identification

Immunostaining

hESC-RPE cells grown on cover-slips were fixed with 4% paraformaldehyde for 15 min, permeabilized using 0.1% Triton X-100 in PBS for 15 min, and blocked for 60 min in 5% goat serum. Primary antibodies against MITF, ZO1, and RPE cell markers including Cralbp, Bestrophin-1 and RPE65 were diluted in the same blocking buffer and incubated with the samples overnight at 4°C, washed, and then incubated with a fluorescently coupled secondary antibody for 1 h at room temperature. The nuclei were stained with 4,6-diamidino-2-phenylindole (DAPI; Invitrogen). Fluorescent images were acquired with a confocal microscope (Zeiss LSM 700, Carl Zeiss; software: Zen lite 2012)[1].

Flow cytometry

Flow cytometry was used to identify the purity of hESC-RPE cells[2-5]. Briefly, cells cultured in growth medium or differentiation medium were detached using HyQTase (Thermo Fisher Scientific) and collected. Cells were blocked with CD32/16 (Bio- Legend) and then incubated for 30 min at 4°C with primary antibodies to surface markers (1:30) or an isotype control (1:30; BioLegend). Cells were rinsed with staining buffer (eBioscience) and a permeabilization buffer (eBioscience) after each step in the procedure. Cells were counted using a FACS Calibur Flow Cytometer and at least 10,000 events were collected for each sample; samples were analyzed using FlowJo software (FlowJo, Ashland, OR, USA).

Real-time PCR analysis

Total RNA was extracted from induced hESC-RPE cells (P3) using TRIzol reagent (9109, TAKARA, Japan) according to the manufacturer's protocol. The eluted RNA was dissolved in sterile DEPC water, and the total RNA concentration was then measured with a spectrophotometer. cDNA synthesis and qPCR following the protocol in the TAKARA user's manual (code no. RR047A). The sequences of the primers used to amplify hRPE cellspecific genes are shown in Supplementary Table S2.

Teratoma formation

100 μl of hESC or hESC- RPE cell suspension (1 × 10^7 cells) was subcutaneously implanted into the groin of SCID mice (regardless of sex)[4]. Mice were maintained under pathogen-free conditions at the animal facility of the Third Military Medical University and received humane care according to the criteria outlined by the requirements of the Laboratory Animal Welfare and Ethics Committee of the Third Military Medical University. We monitored the tumor size and condition of the mice weekly. The animals were anesthetized with overdose three months later and the tissue was examined by a pathologist to identify microscopic pathological changes and evidence of tumor formation.

Clinical Evaluation

mfERG

Multifocal electroretinography (mfERG) elicited by a Veris system (Electro-Diagnostic Imaging, Inc., Burlingame, CA, U.S.A.) was used to test the local function of the macula. All tests were performed according to the International Society for Clinical Electrophysiology of Vision (ISCVE) Standards and repeated three times in every test[6, 7]. The test protocols we used were as follows: the “M-sequence” algorithm was used to control the temporal sequence of change between the light and dark stages of each stimulus hexagon. The stimulus field contained 103 hexagons with a field diameter of 40°. The pupils were fully dilated, bipolar corneal contact electrodes attached, and a real-time fundus camera used to monitor eye movements during the test. Monocular stimulation was used for a four min recording session band-pass filtered at 10-300 Hz. The standard measurement for mfERG amplitude density is the trough-to-peak amplitude (nv) of the N1 and P1 responses over its hexagon in degrees (nv/deg2). Groups of responses from the central to the peripheral retina can be divided into rings 1 to 6.

Microperimetry

All patients underwent four times microperimetry tests before and after surgery[8]. The pupillary was dilated with 0.5% Tropicamide before examination. Exams were performed under dim-light conditions, while the fellow eye was patched. A grid of 45 spots was centered on the fovea

region. The stimulus was a Goldmann III white spot with a stimulus duration of 200 ms. The threshold strategy was HFA 4-2, and background luminance was 31.4 asb. Stimulus attenuation ranged from 0 dB to 34 dB. Total mean retinal sensitivity (MRS) (12° circle), 95 bivariate contour ellipse area (BCEA) (as the area of an ellipse that encompasses 95.4% of fixation points), MRS in the ellipse area, and percentage of fixation points in a circle of 2° (p1) and 4° (p2) diameter were recorded.

Supplemental References:

1. Gao, L., et al., *Intermittent high oxygen influences the formation of neural retinal tissue from human embryonic stem cells.* Sci Rep, 2016. **6**: p. 29944.
2. Osakada, F., et al., *Toward the generation of rod and cone photoreceptors from mouse, monkey and human embryonic stem cells.* Nat Biotechnol, 2008. **26**(2): p. 215-24.
3. Wu, W., et al., *Features specific to retinal pigment epithelium cells derived from three-dimensional human embryonic stem cell cultures - a new donor for cell therapy.* Oncotarget, 2016. **7**(16): p. 22819-33.
4. Li, Q.Y., et al., *Functional assessment of cryopreserved clinical grade hESC-RPE cells as a qualified cell source for stem cell therapy of retinal degenerative diseases.* Exp Eye Res, 2021. **202**: p. 108305.
5. Schwartz, S.D., et al., *Embryonic stem cell trials for macular degeneration: a preliminary report.* Lancet, 2012. **379**(9817): p. 713-20.
6. Hood, D.C., et al., *ISCEV standard for clinical multifocal electroretinography (mfERG) (2011 edition).* Doc Ophthalmol, 2012. **124**(1): p. 1-13.
7. Odom, J.V., et al., *ISCEV standard for clinical visual evoked potentials: (2016 update).* Doc Ophthalmol, 2016. **133**(1): p. 1-9.
8. Serino, F., et al., *Role of Vitreous Detachment in Epiretinal Membrane Peeling: A Multimodal Imaging and Microperimetry Study.* J Clin Med, 2024. **13**(12).
