# Supplementary material for: Safe CNV removal is crucial for successful hESC-RPE transplantation in wet age-related macular degeneration
Source: Stem Cell Reports. 2025 Feb 27;20(3):102424. doi: 10.1016/j.stemcr.2025.102424 (PMC11960522; doi:10.1016/j.stemcr.2025.102424)
Supplement: Document S2. Clinical study protocols [file mmc2.pdf]

Clinical Study Protocols

A preliminary clinical study of local transplantation of human embryonic stem cell-derived retinal pigment epithelial cells for the treatment of macular degeneration

Research Department: Southwest Eye Hospital, Third Military Medical University

Solution version number/date: Version 2.0 / February 5, 2015

Principal Investigator: Yin Zhengqin

Signature:

I. **Introduction**

Macular degeneration, often referred to as age-related macular degeneration (AMD), is the leading cause of blindness in middle-aged and elderly people in Western countries [1]. The loss of acute vision in the center of the macular area seriously affects the ability and quality of independent life of the elderly. Due to the aging of China's population, the number of patients with age-related macular degeneration is increasing, which has become one of the key topics in ophthalmic blindness prevention research. Juvenile macular degeneration (Stargardt's disease) is a less common familial inherited macular degeneration disorder. Patients usually have vision loss from the age of 6-12 years old, and obvious clinical symptoms appear by the age of 20, including blind spots, night blindness, blurred vision, impaired color vision, etc., resulting in countless adolescent patients suffering from vision loss.

According to the clinical and pathological manifestations, age-related macular degeneration is divided into dry and wet types. At present, the main clinical treatment methods are the symptomatic treatment of AMD's wet type, including thermal laser photocoagulation, surgery (excision, macular transposition) and treatment for choroidal neovascularization. In recent years, with the progress of research on the etiology of AMD and the translational medicine research of therapeutic drugs, the social and economic benefits brought by the research have become increasingly significant. The most obvious example is the anti-vascular endothelial growth factor drug used to treat wet age-related macular degeneration, which is very expensive, about 10,000 yuan per treatment, and needs to be repeated every 4 weeks, but

according to multi-center clinical trials, 30% of patients are not sensitive to such drugs, and patients have strong dependence and off-target effects; However, for the vast majority of patients with dry AMD (more than 85% of patients have dry form) and Stargardt disease, there is no clear and effective treatment and fundamental preventive measures.

The pathogenesis of macular degeneration is not fully understood. Existing studies have shown that macular degeneration is closely related to the pathological changes of retinal pigment epithelial cells (RPE) due to genetic or age factors [(2).] RPE cells are monolayer cells whose highly selective transmembrane transport function allows them to play a metabolic role in regulating the choroid and photoreceptors [(3).] In addition, RPE isotic cells are connected as a functional unit and are involved in the formation of vision [(4).] Because RPE in the body does not have the function of self-regeneration and repair, the number of cells decreases with age or due to genetic factors, and the cell function deteriorates. Therefore, some scholars have proposed that RPE cells can be implanted into the subretinal space through in vitro culture through tissue engineering technology to replace the original dysfunctional RPE, so as to save photoreceptor cells and improve vision, which provides a theoretical basis for the clinical treatment of macular degeneration [(5).]

In recent years, with the development and maturity of stem cell culture technology, it has provided a feasible cell source for RPE cell transplantation. At present, there are relatively mature laboratory techniques to obtain stem cells from a variety of cell sources (adult stem cells, embryonic stem cells (ESCs), induced pluripotent stem cells

(iPS), mesenchymal stem cells)[6-10]. Differentiation into RPE cells. A large number of preclinical animal experiments have shown that RPE cell transplantation can play a protective role in retinal optic neuron cells, thereby improving visual function. There have been phase I/II clinical trials in foreign countries to inject human embryonic stem cell-derived RPE cells into the subretinal space for the treatment of advanced AMD and Stargardt disease (NCT01345006, NCT01226628, NCT01344993). Preliminary trial data suggest that this method has a good safety profile and some visual acuity improvement effects[11,12].。 Further clinical trials are underway at multi-center sites in the United States and United Kingdom. This result also announced a breakthrough in the clinical treatment of embryonic stem cells. On September 12 this year, the research team led by Masayo Takahashi, academic leader of RIKEN in Japan, completed the world's first operation to transplant RPE cells made of induced pluripotent stem cells (iPS cells) into the subretinal space of patients with advanced AMD, realizing the clinical application of iPS cells for the first time.

Our group closely follows the international leading level of stem cell therapy for macular diseases, and is currently provided by the Animal Research Institute of the Chinese Academy of Sciences and has been identified by the China Institute for the Control of Pharmaceutical and Biological Products as qualified for clinical treatment The cell line, which has been cultured by our laboratory, has been successfully induced into RPE cells and has reached the standard of clinical application. The results of previous animal experiments showed that in the case model of retinal degeneration RCS rats and retinal photodegenerative pigs, CTS hESC-RPE cells

transplanted into the subretinal cavity played a protective role in optic neurons, and the visual function was also improved. In the clinical trial, in the previous trial of retinal precursor cells and autologous bone marrow mesenchymal stem cells in the subretinal cavity transplantation for the treatment of retinitis pigmentosa or diabetic retinopathy, it was found that the local transplantation of stem cells can improve the function of the diseased retina. In the previous clinical research, our research group has explored a relatively mature surgical plan for local subretinal transplantation, perioperative management of patients, and experience in the use of postoperative immunosuppressants, which has laid a foundation for the clinical application of embryonic stem cells. To sum up, our research group has all the preliminary preparations for the clinical application of embryonic stem cells, and plans to carry out clinical observation and research on embryonic stem cells in the treatment of macular degeneration, so as to fill the domestic gap in the clinical application of embryonic stem cells and reach the international leading level.

2. Purpose of the study

To investigate whether local subretinal transplantation of human embryonic stem cell-derived retinal pigment epithelial cells (ESC-RPE) in patients with macular degeneration has a therapeutic effect and its safety. Exploring new treatment modalities for the clinical treatment of macular degeneration.

3、 What to study

(1) Research design:

This study is a prospective, open-label, self-controlled before-and-after research method

(2) Participate in the study of patient selection

(1) Patients aged 18-65 years (including 18 years old and 65 years old) who have signed the informed consent form

(2) The patient has vision impairment in at least one eye (monocular) or both eyes due to macular degeneration.

(3) Adolescent macular degeneration patients who currently have no effective treatment or age-related macular degeneration (AMD) patients who have been ineffective after 2 treatments with anti-angiogenesis (anti-VEGF) or photodynamic therapy (PDT);

(4) Macular degeneration with voluntary selection of human embryonic stem cell-derived retinal pigment epithelial cells for subretinal space local transplantation.

(5) The study eye must meet the following criteria at follow-up 1:

1) Detected at a distance of 4 meters using the ETDRs visual acuity checklist, the best corrected visual acuity score is between 19 and 73 letters, including 19 and 73 (approximately equivalent to Snellen visual acuity 20/400 to 20/40)

2) The decreased visual acuity in the study eye is due to macular degeneration.

(3) Exclusion criteria

1. Concomitant conditions/diseases of the eye

(1) The study eye has a concomitant disease that would preclude the study treatment from improving visual acuity

(2) Active intraocular inflammation in either eye (whether minor or more severe)

(3) Active infection in either eye (such as conjunctivitis, keratitis, scleritis, ocular pigmentitis, endophthalmitis, etc.)

(4) History of ocular meningitis in either eye

(5) There are diseases in the study eye that may affect the interpretation of the study results or may lead to visual impairment, including severe cataract, glaucoma, retinal vascular occlusion, retinal detachment, macular hole, vitreomacular traction, etc

(6) Iris neovascularization in either eye

(7) The patient is one-eyed or has a best-corrected visual acuity of less than or equal to 24 letters (approximately equivalent to Snellen's visual acuity of 20/320) in the non-study eye (contralateral eye) at follow-up 1

2. Eye treatment

History of intraocular surgery in the study eye

3. General condition or treatment

(1) History of stroke, coronary heart disease, angina pectoris, renal insufficiency requiring dialysis or kidney transplantation, and other systemic chronic diseases that are not suitable for participation in clinical trials

(2) Those who are allergic to sodium fluorescein

(3) Hypertension (systolic blood pressure > 140mmHg or diastolic blood pressure >90mmHg) that cannot be controlled by drugs

(4) Patients with abnormal coagulation function or currently treated with anticoagulant drugs (such as aspirin).

(5) Current or recent systemic use known to be toxic to the lens, retina, or optic nerve may be required

Sexual medications, including chloroquine/hydroxychloroquine, phenothiazine, ethambutol, ferrigin, and tamoxifen, among others

(6) Participated in the clinical study of any investigational drug within 1 month (or 5 half-lives of the investigational drug, whichever is the elderly) before treatment

4. Miscellaneous

(1) Women who have recently (within 6 months) have planned to have children

(2) Pregnant or lactating women

(4) Exit criteria

1. Loss to follow-up

2. In the opinion of the investigator, the treatment is ineffective

3. Occurrence of events that affect the safety of the subject (such as serious adverse events, SAEs, pregnancy, etc.)

(5) Research methods

1. A total of 20 patients with macular degeneration were screened, including 10 cases of juvenile macular degeneration and 10 cases of age-related macular degeneration. Because this study is a preliminary study to explore the efficacy and safety of local subretinal transplantation of human embryonic stem cell-derived retinal

pigment epithelial cells in the treatment of macular degeneration, it is planned to screen 20 patients for observation and study.

2. After the patient is enrolled, complete relevant examinations: visual acuity, optometry, fundus color photo, FFA, OCT, visual electrophysiology, visual field, intraocular pressure, etc.

3. Acquisition, amplification and identification of CTS hESC-RPE. The induction of CTS hESC to RPE, the amplification, purification and identification of CTS-RPE are provided by the GMP Laboratory of the Cell Biotherapy Center of the Southwest Hospital of the Third Military Medical University, which has passed the GMP certification and has the conditions and qualifications for stem cell preparation, see CTS for details hESC-RPE Preparation Technical Operation Standards and Accessories.

4. hESC-RPE treatment: a single subretinal local transplantation of hESC-RPE: 1×106cells.

Schwartz et al.[12] used 5-15×104 hESC-RPE cells for the treatment of macular degeneration (AMD and Stargardt disease) by subretinal transplantation of 5-15104 hESC-RPE cells. According to the results of 2-year follow-up, some patients had a certain improvement in visual function, and none of them had serious adverse events. Preliminary clinical trial results showed no significant difference between different dose groups ($5\times10^{,4}$, 10×, 104, 15× 104 cells). Therefore, we selected 1×106cells as the therapeutic dose for clinical observation.

In the preliminary clinical trials, our group conducted the trial of local transplantation of retinal precursor cells and autologous bone marrow mesenchymal stem cells in the subretinal space for the treatment of retinitis pigmentosa or diabetic

retinopathy. Preliminary experimental results confirm that the surgical method of local subretinal transplantation is safe. In the previous clinical research, our research group has explored a relatively mature surgical plan for local subretinal transplantation, perioperative patient management and postoperative immunosuppressant experience, which has laid the foundation for this clinical study.

5. After treatment, 1 day, 1 week, 4 weeks, 12 weeks, 24 weeks, 36 weeks, and 52 weeks were followed up to check the general condition, visual acuity, optometry, fundus color photo, FFA, OCT, visual electrophysiology, visual field, intraocular pressure, etc.

6. Statistical collation, using its own before and after control to evaluate the efficacy and safety.

(6) Concomitant medication

1. Conventional drugs to delay the development of macular degeneration, and conventional methods for the treatment of macular edema.

2. Antihypertensive drugs: If the patient participating in this clinical study suffers from hypertension, he or she needs to take antihypertensive drugs to control blood pressure.

3. If the patients participating in this clinical study have other systemic chronic diseases, they can take drugs that have no effect on this study.

(7) Evaluation of efficacy

Visual acuity: 2 behaviors are increased and 2 behaviors are reduced, and vision is maintained or improved after treatment.

Improvement of retinal microcirculation: fluorescein fundus angiography

(FFA) was performed for the cases that met the criteria for inclusion in this study, and the FFA contrast time was at least 10 minutes, and the patients were observed to have no perfusion area and neovascularization, and the measurement of the non-perfusion area and neovascular range was measured using the Heidelberg confocal laser fundus scanning computer automatic analysis system to measure the area of the non-perfusion area and the optic disc and find the ratio.

Visual field test: Humphrey 750i automatic perimetry, SITA30-2 program was used to calculate the mean defect of light sensitivity in the central visual field. MD) and corrected patternstandard deviation. CPSD)。 The average value of the light sensitivity dB of 80 loci in the central 30° field of view and 75 loci in the peripheral 60° field of view was calculated, which was used as a statistical test to compare the changes before and after treatment.

OCT inspection: OCT is inspected by Japan Topcon 3D OCT instrument, 8 meridian direction scanning, scanning length 6mm. According to the method of the literature, the distance (thickness) between the inner surface of the retina in the fovea of the macula and the light band of retinal pigment epithelium (RPE) and choroidal capillaries was measured.

Multifocal electroretinogram (mfERG): quantitatively analyzes subtle changes in central visual acuity and local visual function that are imperceptible to routine clinical examination in patients with macular degeneration. The mfERG test was measured using the VERIS Visual Electrophysiology System (EDI United States). The stimulus pattern is an array of 103 hexagons with increasing eccentricity, and the size of the

stimulus field should include a viewing angle of 22.5° on each side of the fixation point. The retinal response was divided into 6 annular regions based on the principle of eccentricity change. Using the central cross as a fixed viewpoint, the monitoring system monitors the location of the image in the fundus and the focus of the image. The amplifier amplification is 100K, the low-frequency cut-off is 3Hz, the high-frequency cut-off is 100Hz, and the reaction sampling frequency is 1200Hz. Before recording, the pupil was dilated to more than 7 mm with compound tropineamide, and the Burian-Allen bipolar contact lens electrode was placed after 1% tetracaine hydrochloride spot ocular surface anesthesia, and the ground electrode was Ag-AgCl discoid skin electrode and placed in the earlobe. Observe the eye area from the computer monitor and adjust the fixation point and refractive focus position.

(8) Security assessment

1. After treatment, 1 day, 1 week, 4 weeks, 12 weeks, 24 weeks, 36 weeks, and 52 weeks were followed up regularly to check the systemic condition and biochemical indicators such as liver and kidney, and to evaluate the safety of hESC-RPE subretinal space local transplantation on the whole body.

2. After treatment, the ocular condition was followed up regularly at 1 day, 1 week, 4 weeks, 12 weeks, 24 weeks, 36 weeks, and 52 weeks to evaluate the safety of allogeneic hESC-RPE subretinal space local transplantation on the eye.

(ix) Informed consent

This study must follow the Declaration of Helsinki and China's relevant GCP clinical trial norms and regulations. Before the start of the study, this research

protocol must be approved by the unit's ethics committee before the trial. Before the patient participates in this study, the study physician should first give a comprehensive introduction to the purpose, procedures and possible risks of this trial in writing or his legal representative, obtain the patient's written consent before implementation, fill in the observation form with the date of consent, and keep the informed consent document signed by the patient for future reference.

(10) Follow-up time and observation indicators:

Number of follow-up visits	1	2(2)	3	4	5	6(3)	7	8
Number of months of study		0		1	3	6	9	12
Number of weeks of study		0	1	4	12	24	36	52
Number of days of study	-14 to -3	1	7（±1）	28（±4）	84（±7）	168（±7）	252（±7）	360（±7）
steps								
Informed consent	×							

medical history	×	×						
Physical examination (4).	×							
Selection/Exclusion Criteria	×	×						
Vital signs	×	×	×	×	×	×	×	×
Laboratory tests (5).	×			×	×	×		×
Best corrected visual acuity	×	×	×	×	×	×	×	×
Eye exam	×	×	×	×	×	×	×	×
Under-eye photo	×			×	×	×	×	×
FFA(1)	×			×	×	×		×
OCT	×			×	×	×	×	×
VEP、ERG	×			×	×	×	×	×
visual field	×			×	×	×	×	×
intraocular	×	×	×	×	×	×	×	×

pressure								
Concomitant medications	×	×	×	×	×	×	×	×
Adverse events	×	×	×	×	×	×	×	×

Note: All × items must be done; Projects marked (×) can be carried out on demand.

FFA: fundus fluorescein angiography; OCT: optical coherence tomography;

(1) At the discretion of the investigator, FFA may be added at any follow-up visit

(2) Follow-up 2 represents baseline

(3) Follow-up 8 represents the end of the study

(4) Physical examination: facial features, chest, lungs, heart, abdomen, pelvis, limbs, nervous system, lymph nodes, skin examination, electrocardiogram, chest X-ray examination, etc

(5) Laboratory tests: blood routine, urine routine, stool routine, liver function, kidney function, coagulation function, hepatitis B three pairs, ICT before blood transfusion, etc

4. Recording and reporting methods of adverse events and adverse reactions

For hESC-RPE subretinal local graft therapy, possible adverse events and management measures include:

1. Infection and fever

2. Local pain in the eye

3. Headache, dizziness, nausea, vomiting reaction

4. Immune rejection and allergic reactions

Dispose:

1. Infection, if there is local redness and swelling or symptoms such as fever and elevated white blood cells in the patient after local transplantation in the subretinal cavity, the infection caused by the local transplantation of the subretinal cavity should be highly suspected, and the blood sample bacterial culture, anti-drug susceptibility test, regular systemic anti-infection treatment with sensitive antibiotics and close observation of the changes in the condition and corresponding treatment.

2. Local pain in the eye: give local hot compress or even drug wet compress, anti-inflammatory, symptomatic and other treatments to relieve the patient's pain. Follow-up observation, if the pain is unbearable, find the cause and actively deal with it.

3. Headache, dizziness, nausea, vomiting and other reactions: According to previous research reports and clinical practice, hESC-RPE transplantation will not cause headache, dizziness, nausea, vomiting and other reactions. If it does, aggressively search for the cause and treat the symptoms.

4. Immune rejection and allergic reactions: hESC-RPE subretinal cavity local transplantation, theoretically, will not have immune rejection and allergic reactions due to the immune immunity environment in the eye. However, due to macular degeneration, the blood-retinal barrier function is impaired, resulting in immune

rejection and allergic reactions after local transplantation of allogeneic hESC-RPE subretinal space, and perioperative and postoperative patients need to take immunosuppressant drugs.

Recently, Lancet published an immunosuppressant method for ES-RPE subretinal transplantation derived from embryonic stem cells, and this study refers to this protocol and integrates the clinically mature immunosuppressant application methods for kidney transplantation.

1) 6 days before surgery;

2) Prednisolone acetate tablets (prednisone): 30mg, oral, once a day, reduce the dose to 15mg after 4 weeks after surgery, oral, once a day, and discontinue 12 weeks after surgery.

3) FK-506: 0.1mg/kg/d, oral, twice a day, check the blood concentration for three days, and maintain it at 10ng/ml. The plasma concentration was maintained at 5 ng/ml, ranging from 3 to 7 ng/ml. (FK506 is packaged at 1mg per tablet), and the drug was discontinued after 12 weeks after surgery.

4) Reference anti-immune rejection dosage after kidney transplantation: Xiaoxi (mycophenolate mofetil capsules, MMF): 500mg orally, 2/day (MMF packaging is 250mg per tablet), and the drug will be discontinued after 4 weeks after surgery.

5) Main side effects of MMF: decreased whole blood, which needs to be done once a week for the first month after medication; Twice a month for the 2nd and 3rd months; Thereafter, a routine blood test should be done once a month. If neutropenia (absolute neutrophil count < 1.3×106/ml), treatment should be interrupted or reduced; Major side effects of FK506: renal impairment, opportunistic infection, renal function and liver function should be rechecked monthly.

The type, degree, occurrence time, duration, treatment measures, and treatment process of adverse events and adverse reactions during the trial should be recorded in the case report form, and their relevance to the treatment regimen should be evaluated on the basis of comprehensive consideration of comorbidities and concomitant medications, and recorded in detail by the investigator.

In addition, when adverse events and adverse reactions are found, the investigator can decide whether to suspend the observation according to the condition, and follow up the cases that stop treatment due to adverse events and adverse reactions, and record the results in detail.

In case of serious adverse events in clinical trials, the investigator should immediately take appropriate protective and therapeutic measures for the subjects, and report to the ethics committee and the research unit in charge (the First Affiliated Hospital of the Third Military Medical University), and the investigator should sign and date the case report form.

5. Protection of the rights and interests of subjects in clinical trials

Ethics committees and informed consent forms are the main measures to safeguard rights. Before the start of the clinical trial, the experimental protocol must be reviewed and approved by the ethics committee and signed before it can be implemented. Any changes to the protocol during the clinical trial should be approved by the Ethics Committee.

The clinical investigator must explain to the subjects that participation in the clinical trial is voluntary, and that they have the right to withdraw from the trial at any

time at any stage of the trial without discrimination or retaliation, and their medical treatment and rights will not be affected, and they can continue to receive other treatments or treatments. Participants must be informed that their participation in the trial and their personal data during the trial are confidential. Participants should also be informed of the nature and purpose of the clinical trial, the expected possible benefits and possible risks and inconveniences, the different groups to which the participants may be assigned to the trial, other treatments available, and the rights and obligations of the participants in accordance with the Helsinki Declaration. Make the subjects fully aware of the clinical trial, give the subjects sufficient time to consider whether they are willing to participate in the trial, and sign the informed consent form.

6. Guarantee of compliance

Taking a variety of measures to improve subject compliance is the key to the success of clinical trials and the authenticity of clinical trial results.

1. First of all, the publicity and education of disease knowledge should be strengthened, so that the subjects have a correct understanding of the disease and medical prevention and treatment knowledge, and when the patients feel that the benefits of treatment outweigh the costs, they will often comply with the doctor's instructions.

2. The investigator explains the details of the clinical trial to the subject, and the informed consent is obtained after fully and in detail explaining the circumstances of the trial.

3. Improve all aspects of health care and enhance adherence, such as: using appointments to improve adherence; Use phone calls or letters to remind you to increase the rate of appointments; Resident doctors for clinical diagnosis and treatment; Arrange the time of the visit reasonably; Re-contact the no-shower; explain the importance of adherence to follow-up and health care education; improving health care services, treatment, etc.

The subject's treatment, follow-up, and reasons for discontinuation of follow-up (e.g., adverse reactions, loss of confidence in treatment, other diseases, etc.) were recorded in detail on the CRF.

7. Data management and statistical analysis

After the end of the clinical trial, the research unit responsible for the management of all clinical case reports, and the establishment of a database for data management and maintenance; SPSS13.0 was used for statistical analysis.

1. Data review and entry: In the process of clinical trials, clinical case observation should be carried out in strict accordance with the clinical trial protocol, the case report form should be filled in on time, and the quality of filling should be ensured. After the clinical study is completed, the quantity and quality of clinical case completions are checked again, and the completeness and accuracy of the data are fully verified and checked before data entry.

2. Statistical analysis: Formulate a statistical analysis plan before statistical analysis of clinical trial data; Clarify the detailed statistical analysis steps and carefully check and confirm the clinical trial data for statistical analysis. The analysis

content was first descriptive statistical analysis, listing the frequency distribution of each variable, and then the efficacy analysis, elimination and dropout case analysis, etc.

8. Summary of data

The unit in charge of the project will summarize and sort out the data analysis results and relevant clinical research data, combine the statistical analysis results with clinical practice to make objective professional conclusions, and write a summary report of the project.

References

1. Lim LS, Mitchell P, Seddon JM, Holz FG, Wong TY. Age-related macular degeneration. Lancet. 2012; 379(9827):1728-38.

2. Zarbin MA. Current concepts in the pathogenesis of age-related macular degeneration. Archives of ophthalmology. 2004; 122(4):598-614.

3. Sparrow JR, Hicks D, Hamel CP. The retinal pigment epithelium in health and disease. Current molecular medicine. 2010; 10(9):802-23.

4. Strauss O. The retinal pigment epithelium in visual function. Physiological reviews. 2005; 85(3):845-81.

5. Binder S, Stanzel BV, Krebs I, Glittenberg C. Transplantation of the RPE in AMD. Progress in retinal and eye research. 2007; 26(5):516-54.

6. Hu J, Bok D. A cell culture medium that supports the differentiation of human retinal pigment epithelium into functionally polarized monolayers. Molecular vision. 2001; 7:14-9.
