# Supplementary material for: Safe CNV removal is crucial for successful hESC-RPE transplantation in wet age-related macular degeneration
Source: Stem Cell Reports. 2025 Feb 27;20(3):102424. doi: 10.1016/j.stemcr.2025.102424 (PMC11960522; doi:10.1016/j.stemcr.2025.102424)
Supplement: Document S3. Article plus supplemental information [file mmc4.pdf]

Stem Cell Reports
Article
OPEN ACCESS

Safe CNV removal is crucial for successful hESC-RPE transplantation in wet age-related macular degeneration

Ying Xue Lv,[1,2,3,5] Qi You Li,[1,2,3,5] Ping Duan,[1,2,3] Min Fang Zhang,[1,2,3] Bo Liu,[1,2,3] Shi Ying Li,[4] Tong Tao Zhao,[1,2,3,*] Hao Wang,[1,2,3,*] Yong Liu,[1,2,3,6,*] and Zheng Qin Yin[1,2,3]

[1]Southwest Hospital/Southwest Eye Hospital, Third Military Medical University (Army Medical University), Chongqing, China
[2]Key Lab of Visual Damage and Regeneration & Restoration of Chongqing, Chongqing, China
[3]Jinfeng Labatory, Chongqing, China
[4]Department of Ophthalmology, the First Affiliated Hospital of Xiamen University, School of Medicine, Xiamen, Fujian Province, China
[5]These authors contributed equally
[6]Lead contact
*Correspondence: doct_ztt@163.com (T.T.Z.), wanghao4295@foxmail.com (H.W.), liuyong@tmmu.edu.cn (Y.L.)
https://doi.org/10.1016/j.stemcr.2025.102424

SUMMARY

Subretinal transplantation of human embryonic stem cell-derived retinal pigment epithelial (hESC-RPE) cells has demonstrated therapeutic potential in macular degeneration. However, its efficiency is limited in wet age-related macular degeneration (wet AMD) due to choroidal neovascularization (CNV). To investigate the feasibility of hESC-RPE cell transplantation, we employed a surgical approach to induce retinal detachment, which allowed the removal of CNV lesions. After retinal reattachment, hESC-RPE cells were transplanted into the subretinal space. Ten patients were enrolled and divided into 2 groups. No retinal edema or CNV recurrence was observed in group 1 (7 patients without bleeding). Group 2 (3 patients with bleeding) had persistent fundus inflammation, and one patient experienced CNV recurrence. All patients were managed effectively without vision loss. These findings suggest that subretinal transplantation of hESC-RPE cells after CNV removal is safe and well tolerated; however, damage caused during CNV removal may trigger persistent inflammation and CNV recurrence. This study was registered at ClinicalTrials.gov (NCT02749734).

INTRODUCTION

Age-related macular degeneration (AMD) is the leading cause of vision loss in the elderly worldwide. Advanced AMD is characterized by damage to the retinal pigment epithelium (RPE), Bruch's membrane, and choroidal capillaries, leading to progressive visual impairment. AMD can be classified as macular neovascularization (wet AMD) and geographic atrophy (dry AMD), both of which ultimately result in a significant loss of visual function (Friedman et al., 2004; Wong et al., 2014). While anti-vascular endothelial growth factor (anti-VEGF) therapy has achieved substantial success in managing wet AMD, effective treatment is still needed for the late-stage form, which is characterized by submacular neovascularization or disciform scar.

Advanced Cell Technology Inc. pioneered the first human embryonic stem cell (hESC)-derived RPE transplantation for dry AMD and Stargardt disease, which yielded promising outcomes (Schwartz et al., 2012, 2015). Subsequent studies have suggested that subretinal transplantation of RPE may offer new hope for the treatment of non-exudative macular degeneration (da Cruz et al., 2018; Kashani et al., 2018; Mandai et al., 2017; Song et al., 2015). However, treating wet AMD with RPE transplantation remains challenging because of the presence of submacular neovascularization, disruption of the blood-retinal barrier (BRB), and associated inflammation or hemorrhage (Deng et al., 2022; Spraul et al., 1999).

Surgical removal of submacular neovascular membranes in wet AMD was first attempted decades ago (Merrill et al., 1999; Thomas et al., 1992). Although the procedure often achieved anatomic success, visual outcomes were suboptimal. This was largely due to the inevitable removal of RPE cells situated on the surface of the neovascular complex (Grossniklaus and Gass, 1998) and macular injuries caused by the use of retinotomy sites near the fovea and laser photocoagulation.

Recently, studies have explored subretinal transplantation of hESC-RPE or autologous induced pluripotent stem cell-derived RPE (iPSC-RPE) in wet AMD, with or without choroidal neovascularization (CNV) removal. A team from RIKEN reported a case of autologous iPSC-RPE sheet transplantation after CNV removal. Although structural recovery was achieved, visual function did not improve, possibly due to the autologous nature of iPSC-RPE carrying pathogenic mutations (Mandai et al., 2017; Takagi et al., 2019). Similarly, da Cruz et al. (2018) and Sugita et al. (2020) reported hESC-RPE transplantation (as a patch or cell suspension) in wet AMD without CNV removal. In these studies, the persistence of the CNV membrane compromised the survival of the transplanted cells and hindered the recovery of the macular structure. This highlights the importance of using allogeneic grafts devoid of

© 2025 The Authors.
Published by Elsevier Inc. on behalf of International Society for Stem Cell Research.
This is an open access article under the CC BY license (http://creativecommons.org/licenses/by/4.0/).

pathogenic genes and the necessity of CNV membrane removal for successful RPE transplantation in wet AMD.

In a previous study, we reported three cases of subretinal transplantation of hESC-RPE in wet AMD. Peripheral retinotomy was performed to safely and thoroughly remove the CNV membrane, avoiding inadvertent damage to the macular fovea and preventing the transplanted cells from diffusing into the vitreous cavity. In all three patients, the procedure demonstrated good safety and tolerability and improved or stabilized vision.

In the present study, we explored the clinical applications of subretinal hESC-RPE transplantation for wet AMD. Our findings underscore the importance of safe CNV membrane removal to ensure the safety and efficacy of transplantation. Herein, we report these results in detail.

RESULTS

Safety of one-year postoperative course after transplantation of hESC-derived RPE

Ten patients with wet AMD (6 males and 4 females; average age, 61; range 48–71 years; Table 1) were enrolled. Seven patients previously underwent intraocular anti-VEGF treatment but developed a submacular fibrovascular membrane, leading to progressive visual decline. Nine patients with active CNV lesions presented with macular edema, subretinal hemorrhage, or leakage on fundus fluorescein angiography (FFA) preoperatively (Table 1). Preoperative best-corrected visual acuity (BCVA) ranged from hand motion (near blindness, 0 Early Treatment Diabetic Retinopathy Study [ETDRS] letters) to 20/400 (severe vision loss, 15 ETDRS letters), as summarized in Table 1.

Complete removal of the fibrous membrane was achieved in seven patients, while 3 patients (patients 8, 9, and 10) experienced fibrovascular hemorrhage during CNV removal. The fibrovascular membrane was partially shaved in 1 of the 3 cases, leaving some residual CNV. Postoperatively, no severe complications such as endophthalmitis, secondary glaucoma, or retinal detachment (RD) occurred. The characteristics of all patients who completed the study are summarized in Table 1. None of the patients exhibited adverse systemic effects or uncontrolled proliferation of transplanted cells throughout the follow-up period.

Three months after surgery, 7 patients (patients 2, 3, 4, 6, 8, 9, and 10) developed epiretinal membrane (ERM), and silicone oil removal combined with pre-macular membrane stripping was performed. Five patients (patients 2, 6, 8, 9, and 10) developed late macular edema, which gradually resolved after local anti-inflammatory therapy, except for 1 patient (patient 10) who experienced persistent edema at 9 months. Optical coherence tomography angiography (OCTA) indicated CNV recurrence, and the patient received anti-VEGF treatment, resulting in the resolution of macular edema.

The changes in visual acuity of all patients are presented in Table 1 and Figure 1. Overall, postoperative BCVA either improved or was maintained without a significant decline in visual acuity. Compared with baseline, BCVA improvement, defined as an increase of >10 ETDRS letters, was observed in 6 of 10 patients (60%; patients 1, 2, 4, 5, 6, and 10) at 1 year postoperatively, with 3 patients (30%; patients 2, 5, and 6) showing an increase of >20 letters. Of the remaining 4 patients, 3 (30%; patients 3, 8, and 9) had stable visual acuity after surgery, fluctuating around 5 letters. One patient (patient 7) initially showed significant improvement at 6 and 9 months but experienced a sharp decline to below baseline at 12 months. As shown in Table 1, postoperative adverse events, such as macular edema, occurred in all 3 patients with intraoperative hemorrhage, suggesting that intraoperative bleeding and residual CNV may have negatively affected hESC-RPE cell survival. For further investigation, we divided the 10 patients into two groups: group 1, without intraoperative bleeding (7 patients), and group 2, with intraoperative bleeding (3 patients). Group 1 showed a lower incidence of macular edema (2/7 vs. 3/3) and ERM (4/7 vs. 3/3) than group 2 (Table 1). Visual acuity improvement was greater in group 1 (average increase of 12 letters) than in group 2 (average increase of 3 letters) (Figure 1).

Structural and functional recovery in group 1

Seven patients (patients 1–7) in group 1 demonstrated significant structural and functional recovery after hESC-RPE cell transplantation. The fibroproliferative membrane was successfully removed without intraoperative submacular hemorrhage. The subretinal fluid bleb that formed was clinically resorbed within 24 h, with no cases of persistent or unintended RD. A stable RPE-like cell layer formed at the lesion site, as confirmed by spectral domain optical coherence tomography (SD-OCT), with no signs of recurrent CNV or retinal inflammation.

For example, patient 1 showed complete resolution of the preoperative hyperreflective mass and intraretinal cysts postoperatively, with no recurrence of hemorrhage or subretinal pigmentation (Figures 2A3, 2B2–2B4, and 2C1). One month after surgery, hyperreflective punctate signals began to appear on the bare Bruch's membrane in the area where the lesion had been removed and appeared devoid of native RPE. After three months, these punctate signals gradually developed into a new cell mass attached to the host retina, resembling the native RPE outside the graft region. Additionally, the relative preservation of the outer nuclear layer (ONL) corresponded to this cell mass

Table 1. Characteristics and clinical information of patients with wet AMD

Group	Number	Sex	Age (years)	History	Operated eye	Preop anti-VEGF drugs	Characteristics at screening: Macular edema	Characteristics at screening: Leakage on FFA	Characteristics at screening: Hemorrhage	Visual acuity by ETDRS: Baseline	Visual acuity by ETDRS: 1 m follow-up	Visual acuity by ETDRS: 3 m follow-up	Visual acuity by ETDRS: 6 m follow-up	Visual acuity by ETDRS: 9 m follow-up	Visual acuity by ETDRS: 12 m follow-up	Complications: Hemorrhage	Complications: Residual CNV	Complications: Recurrent CNV	Complications: Macular edema: Early	Complications: Macular edema: Late	Complications: ERM
1	P-001	M	62	1 year	OD	yes	✓	✓	✓	0	41	38	13	16	13	–	–	–		–	–
	P-002	F	60	2 years	OS	yes	✓	✓		10	24	19	19	29	36	–	–	–	✓	✓	✓
	P-003	M	68	10 years	OD	no	✓	✓	✓	15	12	14	14	15	19	–	–	–	–	–	✓
	P-004	F	71	4 months	OD	no	✓	✓	–	5	12	7	8	8	16	–	–	–	–	–	✓
	P-005	M	48	1 year	OS	yes	✓	✓	–	0	0	2	15	4	23	–	–	–	–	–	–
	P-006	M	59	2 years	OS	yes	✓	✓	–	0	7	4	12	23	35	–	–	–	–	✓	✓
	P-007	M	62	2 years	OD	yes			–	15	9	15	35	33	9	–	–	–	–	–	–
2	P-008	M	70	6 years	OS	yes	✓	✓	–	5	0	0	1	1	2	✓	–	–	✓	✓	✓
	P-009	F	65	1 year	OS	no	✓	✓	–	11	10	15	15	13	14	✓	–	–	✓	✓	✓
	P-010	F	51	8 years	OS	no	✓	✓	–	12	10	12	8	8	22	✓	✓	✓	✓	✓	✓

AMD, age-related macular degeneration; hESC-RPE, human embryonic stem cell-derived retinal pigment epithelium; P, patient; F, female; M, male; OD, right eye; OS, left eye; Preop, preoperative; VEGF, vascular endothelial growth factor; FFA, fundus fluorescein angiography; ETDRS, Early Treatment Diabetic Retinopathy Study (ETDRS) visual acuity test (best-corrected visual acuity); m, month; CNV, choroidal neovascularization; ERM, epiretinal membrane.

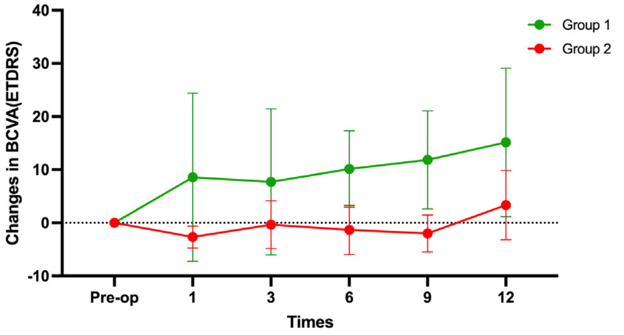

Figure 1. Changes in the BCVA over time of the operated eyes
BCVA was recorded preoperatively and at 1, 3, 6, 9, and 12 months postoperatively. Group 1 showed a progressive improvement in BCVA, while group 2 demonstrated relatively stable or slight decreases from 3 to 9 months after surgery. Both group 1 and group 2 patients achieved visual improvement at 12 months follow-up, but the visual outcome in group 1 was better than that in group 2. Note that group 2's visual acuity decreased to a certain extent, but it improved after appropriate treatment. Green line: group 1 (patients 1–7); red line: group 2 (patients 8–10).

structure. One year later, structural recovery persisted (Figure 2C3). Furthermore, no recurrent active CNV, additional vascular leakage, or local inflammation was observed at the graft site, as shown by FFA and indocyanine green angiography (ICGA) imaging (Figure 2C2). BCVA improved by 41 letters one month postoperatively and remained 13 letters higher than the preoperative baseline with significance (Table 1).

Patient 2 also developed a new cell mass formation at the lesion site (Figure S2D3). However, postoperative irregular ERM led to retinal traction and edema 1 month after surgery. Mild vascular leakage was observed during the late phase of FFA (Figure S2C3). After silicone oil removal and pre-macular membrane peeling at 3 months, the fluorescence leakage resolved, and all intraretinal fluid was absorbed (Figure S2D2 and S2D3). BCVA was improved by 36 letters at the last follow-up (Table 1).

At 12 months, 5 of 7 patients in group 1 had significant vision improvement, with 2 patients gaining >20 letters, all without the need for additional anti-VEGF injections after surgery. Two patients maintained their baseline vision but experienced transient improvement earlier during the follow-up period. Patient 7 initially had a BCVA of 15 ETDRS letters in the treated eye, which improved to 35 letters by 6 months postoperatively but sharply declined to 9 letters by the last visit (Table 1). Microperimetry showed improvement from baseline at 3–6 months after transplantation but regressed to baseline at 12 months. The multifocal electroretinogram (mfERG) showed no significant change during follow-up (Figures S3–S6).

Persistent macular edema and CNV recurrence in group 2

Group 2 patients (those with intraoperative bleeding; patients 8, 9, and 10) developed a more chaotic subretinal structure, showing a mixture of blood, transplanted cells, and residual CNV. Persistent macular edema was observed in 2 patients (patients 8 and 9) due to unresolved inflammation, while 1 patient had CNV recurrence (patient 10). Treatment was individualized based on the underlying cause of the complications: either inflammation or CNV recurrence.

For example, patient 8 presented with a typical CNV lesion preoperatively, as revealed by ICGA and OCTA imaging (Figures 3A2 and 4A1). During surgery, hemorrhage occurred when removing the CNV (Figure 3B2 and 3B3). After removing the blood, we completed the procedure and subsequently transplanted hESC-RPE cells into the subretinal space (Figure 3B4). One month after surgery, a small amount of submacular bleeding was observed, as well as minor fluorescence leakage on FFA (Figures 3C1 and 3C2), suggesting that CNV removal may have caused structural damage to the retina and Bruch's membrane. At the 3- and 6-month follow-ups, patient 8 experienced persistent macular edema despite immunosuppressive and local anti-inflammatory therapies (Figure 3D3). Increased fluorescence leakage was observed in the late phase of FFA (Figure 3D2), although no neovascularization was detected on OCTA (Figure 4D2). This indicated that macular edema was not caused by CNV recurrence but was likely due to a chronic inflammatory response. Given the disruption of the BRB, we suspect that immune rejection was inevitable and was the cause of the prolonged edema despite normal systemic blood tests, including peripheral blood T cell/T cell subclass counts and cytokine levels. Treatment with oral immunosuppressants and intravitreal triamcinolone acetonide (IVTA) eventually resolved the edema (Figure 3E3), and the patient's vision remained stable compared with preoperative levels. The clinical manifestations and management of patient 9 were similar to those of patient 8. Persistent macular edema was the primary issue, with FFA showing fluorescence leakage and OCTA revealing no neovascularization, suggesting chronic postoperative inflammation.

Patient 10 presented preoperatively with relatively dense CNV and close adhesion to the choroid, making complete removal challenging (Figure 5A3). To minimize the risk of uncontrollable bleeding and structural damage, as much CNV tissue as possible was removed, although a small amount remained (Figure 5B3). One month after surgery, the macular structure had recovered well; however, by the 3-month follow-up, significant macular edema had developed (Figure 5D3). FFA showed slight late-phase fluorescence leakage, and the macular edema did not improve

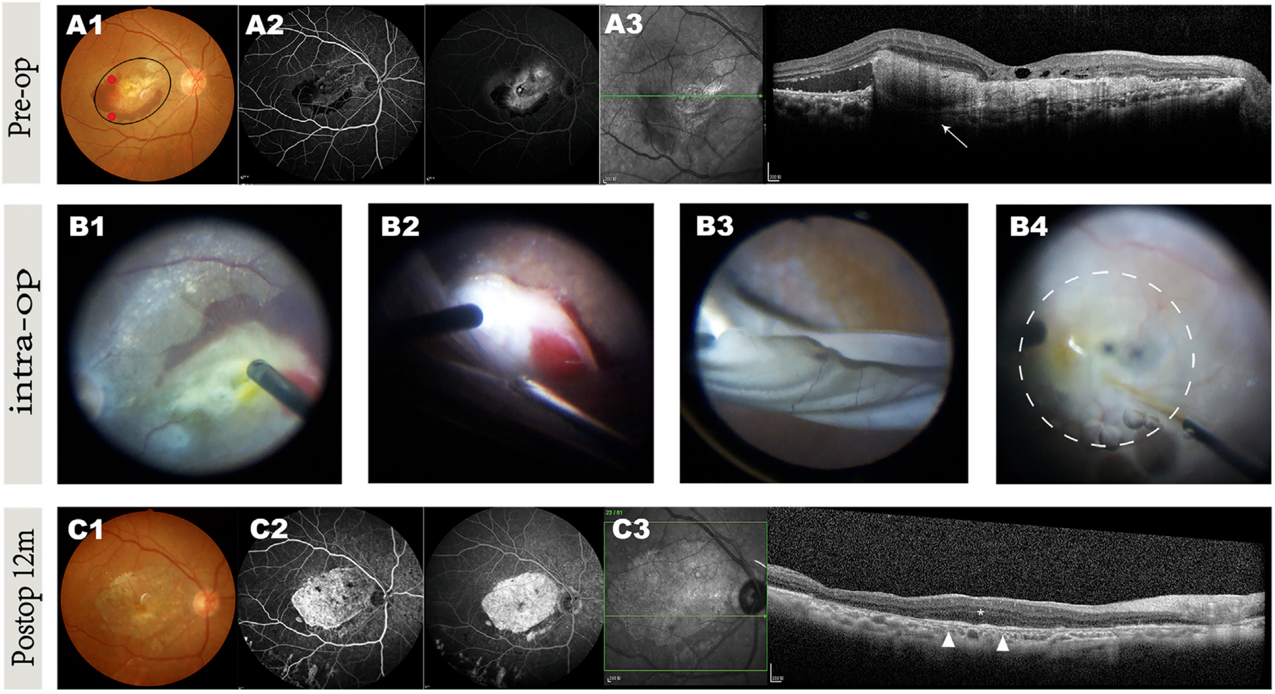

Figure 2. Retinal morphological changes in the operated eye of patient 1
Retinal examination before and after CNV removal coupled with hESC-derived RPE suspension transplantation, at preoperative (A1–A3), intraoperative (B1–B4), and postoperative (C1–C3) stages.
(A) Preop (A1–A3): (A1) fundus photography showing the fibrous membrane and the extensive paramacular submacular hemorrhage secondary to wet AMD. The injection site is marked by red dots, and the formed subretinal bleb is indicated by a black circle. (A2) FFA images highlighting the obscured shape of subfoveal CNV due to the hemorrhage (early phase) and the leakage with hyperfluorescence and areas of nonperfusion (late phase), suggesting the lesion was active. (A3) SD-OCT scan shows a hyperreflective mass of retinal edema and subretinal deposits (arrow). The signal beneath the CNV was hypo-transmission.
(B) Intraop (B1–B4): (B1) intraoperative image before incising the peripheral temporal quadrant retina. (B2) After incising the retina, the fiber membrane is visible and easy to peel off without choroidal vessel damage. (B3) No fundus hemorrhage was observed after complete removal of the CNV membrane. (B4) A subretinal bleb was formed after subretinal injection of transplanted cells, marked by a dashed circle.
(C) Postop 12 months (C1–C3): (C1) fundus photo showing no subretinal pigmentation, hemorrhage, or recurrent CNV, but macular geographic atrophy had developed. (C2) FFA with expanded transmitted-fluorescence area and no fluorescence leakage. (C3) SD-OCT image demonstrating complete removal of CNV and the hyperreflective cell layer-like structure (arrowheads) over the bare but almost intact Bruch's membrane, with excellent restoration of the outer nuclear layer (ONL, asterisk) compared to adjacent regions.

despite continuous anti-inflammatory therapy (Figure 5E3). Although the initial postoperative recovery was promising, CNV recurrence was detected at 6 months using OCTA (Figure 6D2), which led to further macular edema. After 3 consecutive anti-VEGF injections, the macular edema resolved (Figure 5F3), and the patient's visual acuity improved by 22 letters from the baseline (Table 1). Representative images of all patients are shown in the supplemental information (Figures S3–S6).

DISCUSSION

In this study, we confirmed the safety and tolerability of transplanted hESC-RPE cells in the subretinal space when the CNV lesions were removed without complications. We found that safe CNV removal is a prerequisite for successful hESC-RPE transplantation in patients with wet AMD. Close adhesion of the CNV to the choroid resulted in intraoperative bleeding and persistent inflammation or CNV recurrence. Therefore, a safe method for CNV removal was designed.

In the 1990s, ophthalmologists used temporal macular retinotomy to remove CNVs. This technique involved using a surgical pick to disconnect the CNV and choroid through a small incision adjacent to the macula (Thomas et al., 1992). However, this approach poses a significant risk of injuring the macula and choroid surrounding the CNV. In this study, we performed a peripheral retinotomy and flipped the detached temporal retina toward the nasal

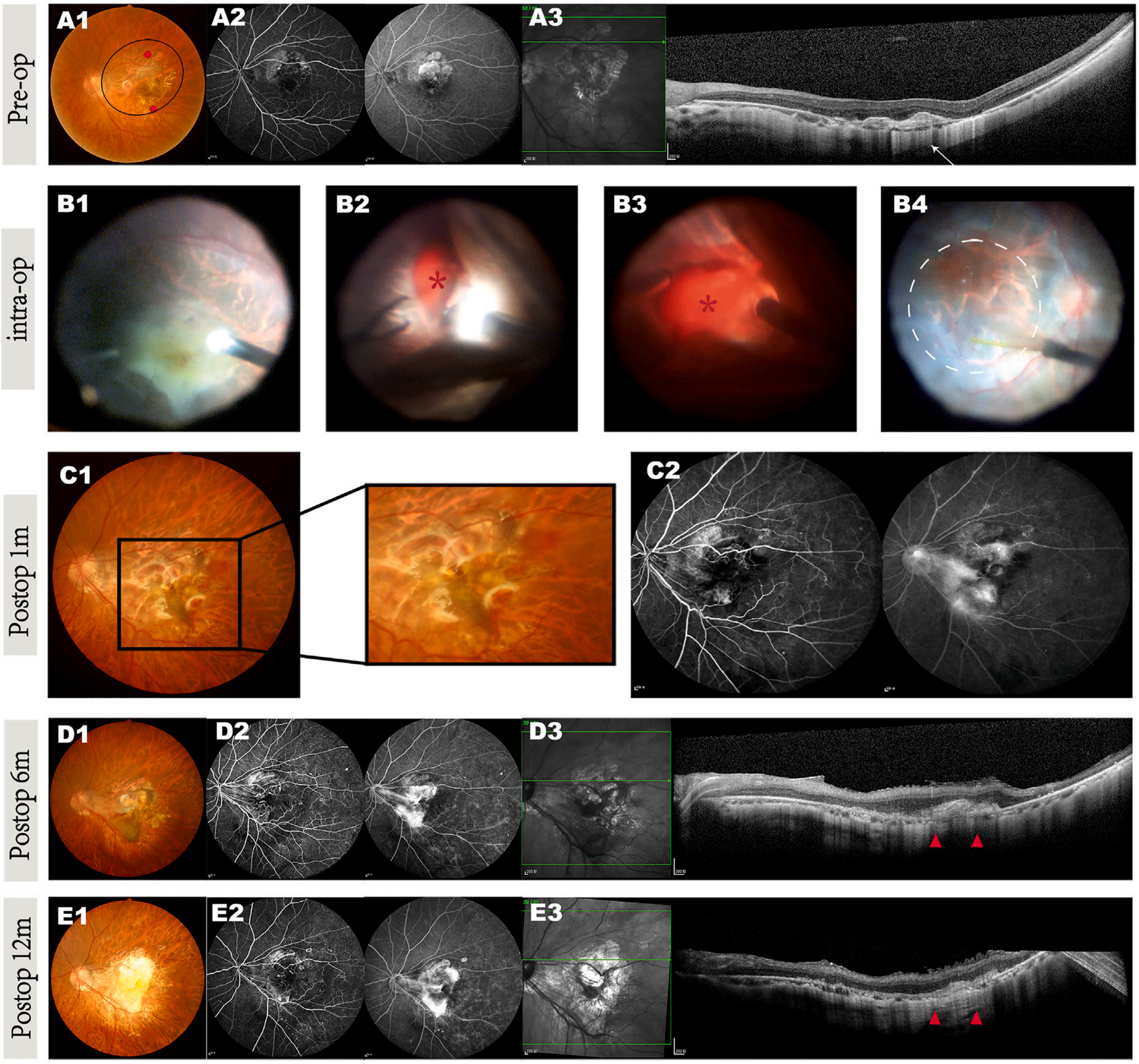

Figure 3. Retinal morphological changes in the operated eye of patient 8

Retinal examination before and after CNV removal coupled with hESC-derived RPE suspension transplantation; preoperative (A1–A3), intraoperative (B1–B4), and postoperative images at 1 month (C1 and C2), 6 months (D1–D3), and 12 months (E1–E3) are shown.

(A) Preop (A1–A3): (A1) fundus photography showing the fibrovascular disciform scar and pigment changes in the foveal area. The injection site is marked by red dots, and the formed subretinal bleb is indicated by a black circle. (A2) FFA images highlighting the typical CNV shapes without obvious late fluorescence leakage. (A3) SD-OCT scan showing fibrotic neovascular membrane breaks (arrow) through the RPE layer. The signal beneath the CNV was hyper-transmission.

(B) Intraop (B1–B4): (B1) intraoperative image before incising the peripheral temporal quadrant retina. (B2) After incising the retina, the fiber membrane was tightly adhered, and hemorrhage occurred during the membrane peeling process (asterisk). (B3) Significant fundus hemorrhage was observed (asterisk) and subsequently cleaned after complete removal of the CNV membrane. (B4) A subretinal bleb (dashed circle) formed after subretinal injection of transplanted cells. A small amount of subretinal blood remains visible in the macula.

(C) Postop 1 month (C1 and C2): (C1) the blood is still present, and pigmentation has appeared at the original lesion site on fundus photography. (C2) A small amount of fluorescence leakage is visible at the nasal side of the macula, which intensified at the late phase.

(legend continued on next page)

side of the optic disc. This method directly exposes the subretinal CNV membrane to the surgeon, thereby providing ample space for a precise excision. In addition, this procedure minimizes damage to the macular neuroretina (e.g., macular hole) and unnecessary RPE-choroid injury (hemorrhage) by offering a sufficient surgical field of view and facilitating the use of both hands for the procedure. As a result, neither macular holes nor uncontrolled choroidal hemorrhages occurred in this study. Postoperatively, some patients developed ERM, likely due to proliferative vitreoretinopathy (PVR). However, no serious complications such as RD occurred. The ERM was successfully removed during the silicone oil removal surgery, which was performed 3 months after transplantation.

Since Matthew Thomas first reported CNV excision in wet AMD in 1992 (Thomas et al., 1992), at least 31 studies on this surgical approach alone were published between 1992 and 2004 (Falkner et al., 2007). Some studies have suggested that CNV excision might provide potential benefits compared to the natural progression of CNV in wet AMD (Guthoff and Schrader, 2004; Merrill et al., 1999). However, in 2004, the Submacular Surgery Trials (SST) research group performed two large randomized clinical trials comparing surgical removal with observation (Bressler et al., 2004; Hawkins et al., 2004). Both trials concluded that CNV excision did not significantly improve visual acuity in patients with wet AMD. Although surgery effectively removes CNV, it also results in an unavoidable loss of RPE cells in the macular area. RPE loss is particularly detrimental to the functional recovery of the macular photoreceptors, severely limiting visual restoration after surgery. Therefore, an alternative source of RPE cells is required to support the recovery of macular function.

In previous studies of RPE transplantation for wet AMD, various forms of RPE cells were utilized, including autologous RPE-choroid patches, iPSC/hESC-derived RPE patches, and iPSC/hESC-derived RPE suspensions (Alexander et al., 2015; da Cruz et al., 2018; Mandai et al., 2017; Mehat et al., 2018; Sugita et al., 2020). Autologous RPE-choroid patches, once a viable option, caused significant damage to the peripheral retina and choroid and were no longer considered a mainstream treatment. iPSC/hESC-derived RPE patches demonstrated a more robust monolayer RPE structure and higher cell survival rates in subretinal transplantation (Diniz et al., 2013). However, RPE cell death within the transplanted patch often resulted in localized cell defects because of the single-layer structure. Furthermore, the prolonged preparation time required for RPE sheet transplantation posed the additional risk of irreversible visual damage (Uppal et al., 2007). Although RPE suspension transplantation is associated with lower cell survival rates and challenges in forming a monolayer RPE structure, it offers a significant advantage in that it allows for the transplantation of a large number of cells. Although there is a risk of RPE cell suspension leakage into the vitreous cavity, a large peripheral retinotomy facilitates safer CNV removal, minimizes macular neuroretina damage, and reduces the likelihood of RPE cell leakage. These surgical strategies proved effective as postoperative examinations revealed no evidence of transplanted cells in the vitreous cavity or anterior chamber, confirming the success of the procedure.

In group 1, CNV removal was safe and successful. A cell-like structure formed in the RPE transplantation area and may support the surrounding retinal structure. Morphologically, we observed the transplanted cells as a new cell-like mass in the transplantation area on SD-OCT, forming a layered structure in some patients (Figure 2C3 of patient 1). There was no sign of an inflammatory reaction, such as subretinal fluid or fluorescence leakage on FFA or ICGA. In addition, the ONL within the new cell mass area showed relatively good restoration compared with the regions outside the graft, indicating that the transplanted cells may have survived and provided support to the host retina. Functionally, BCVA improved in some patients and remained stable in others during the follow-up period; the mfERG and microperimetry were also stable. These findings are consistent with those of preclinical (Lu et al., 2009; Wang et al., 2008) and clinical reports (da Cruz et al., 2018; Mehat et al., 2018; Song et al., 2015). This indicated that the transplanted RPE cells protected the macular photoreceptors and promoted functional recovery. In this group, the incidence of postoperative ERM formation and macular edema was low, and visual function improvement was more favorable.

Group 2 patients faced greater challenges in macular structure restoration due to the complex nature of CNVs and intraoperative bleeding, which led to persistent inflammation and macular edema. A review of the surgical videos for group 2 revealed that the fibroproliferative membrane was difficult to remove because of its close adhesion to the choroid. This removal process caused damage to the

(D) Postop 6 months (D1–D3): (D1) the pigmentation area at the injection site has expanded. (D2) FFA images showing increased fluorescence leakage in the late phase, observed after ERM peeling at 3 months. (D3) Retinal edema persists with subretinal deposits (arrowheads).

(E) Postop 12 months after oral immunosuppressants and intravitreal TA (Triamcinolone Acetonide) injections (E1–E3): (E1) subretinal hemorrhage and pigmentation have disappeared. (E2) Fluorescence leakage has subsided. (E3) The retinal edema has resolved (arrowheads).

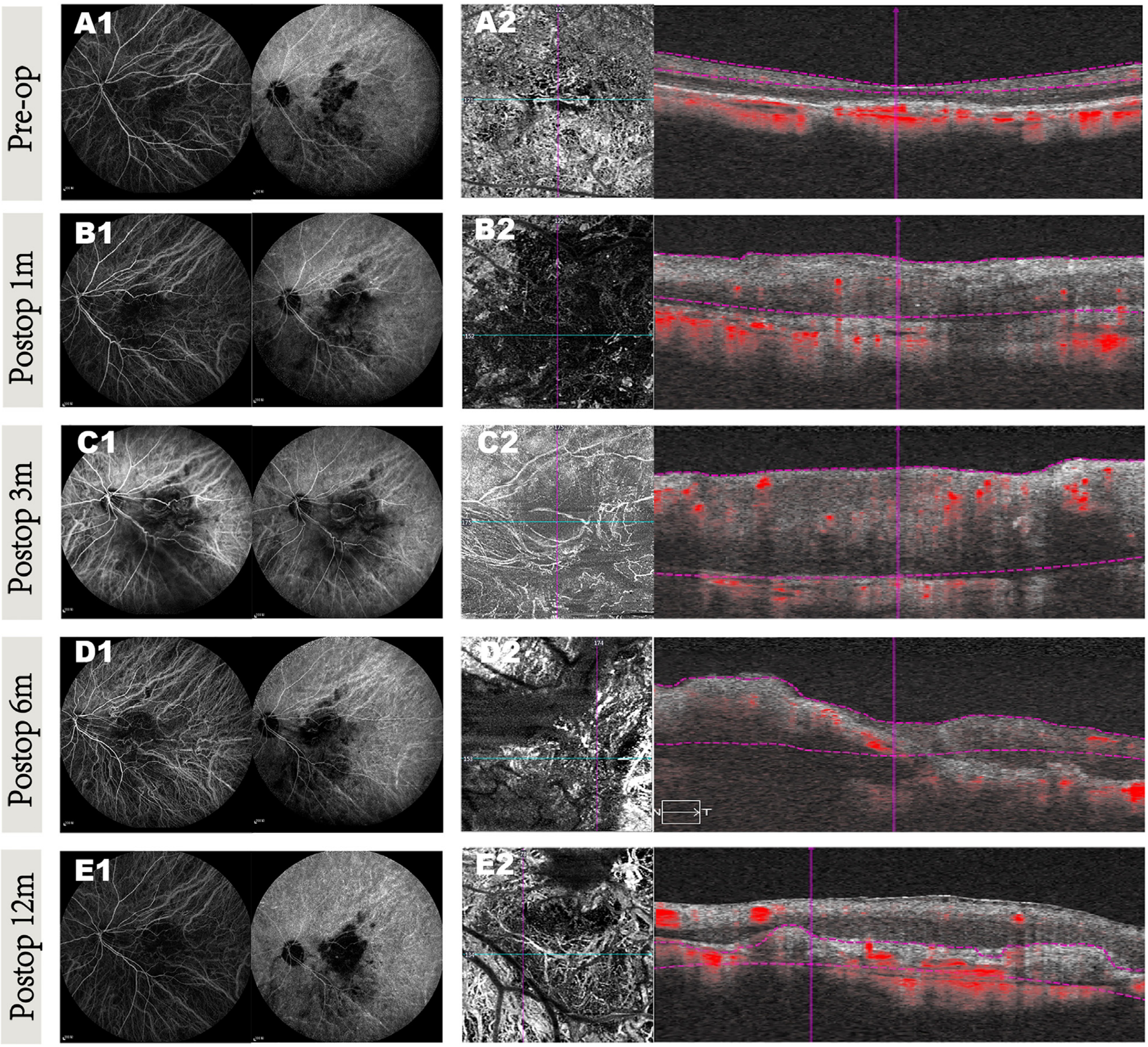

Figure 4. CNV changes before and after surgery over a 12-month period in patient 8

(A) Preop (A1 and A2): (A1) early (left side) and late (right side) phase of ICGA images. (A2) OCTA images reveal significant choroidal neovascularization blood signal in the macular area preoperatively.

(B–E) Postoperative images at 1 month (B1 and B2), 3 months (C1 and C2), 6 months (D1 and D2), and 12 months (E1 and E2): after surgery, ICGA images (C1, D1, and E1) and OCTA images (C2, D2, and E2) show progressive reduction in macular edema, with continued retinal architecture improvement and decreased hyperreflective material over the 12-month follow-up. There was no blood signal from residual (B2) or recurrent choroidal neovascularization (C2–E2).

choroidal vessels, resulting in significant hemorrhage that took >1 month to resolve, and part of the CNV could not be completely removed. Hemorrhage and residual CNV resulted in BRB damage and failure of ocular immune privilege (Zhou and Caspi, 2010). Therefore, we observed macular edema on OCT, fluorescence leakage on FFA/ICGA, and CNV recurrence on OCTA in this group. The most likely cause of the apparent inflammatory response in these patients was immune rejection owing to BRB damage. Residual CNV was the direct cause of CNV recurrence. For example, in patient 10, FFA and ICGA showed obvious fluorescence leakage due to immune rejection (Figure 5D2). OCTA revealed residual CNV (Figure 6B2), and the residual CNV grew rapidly until relapse.

The treatment regimens were adjusted based on whether the patient experienced CNV recurrence. The

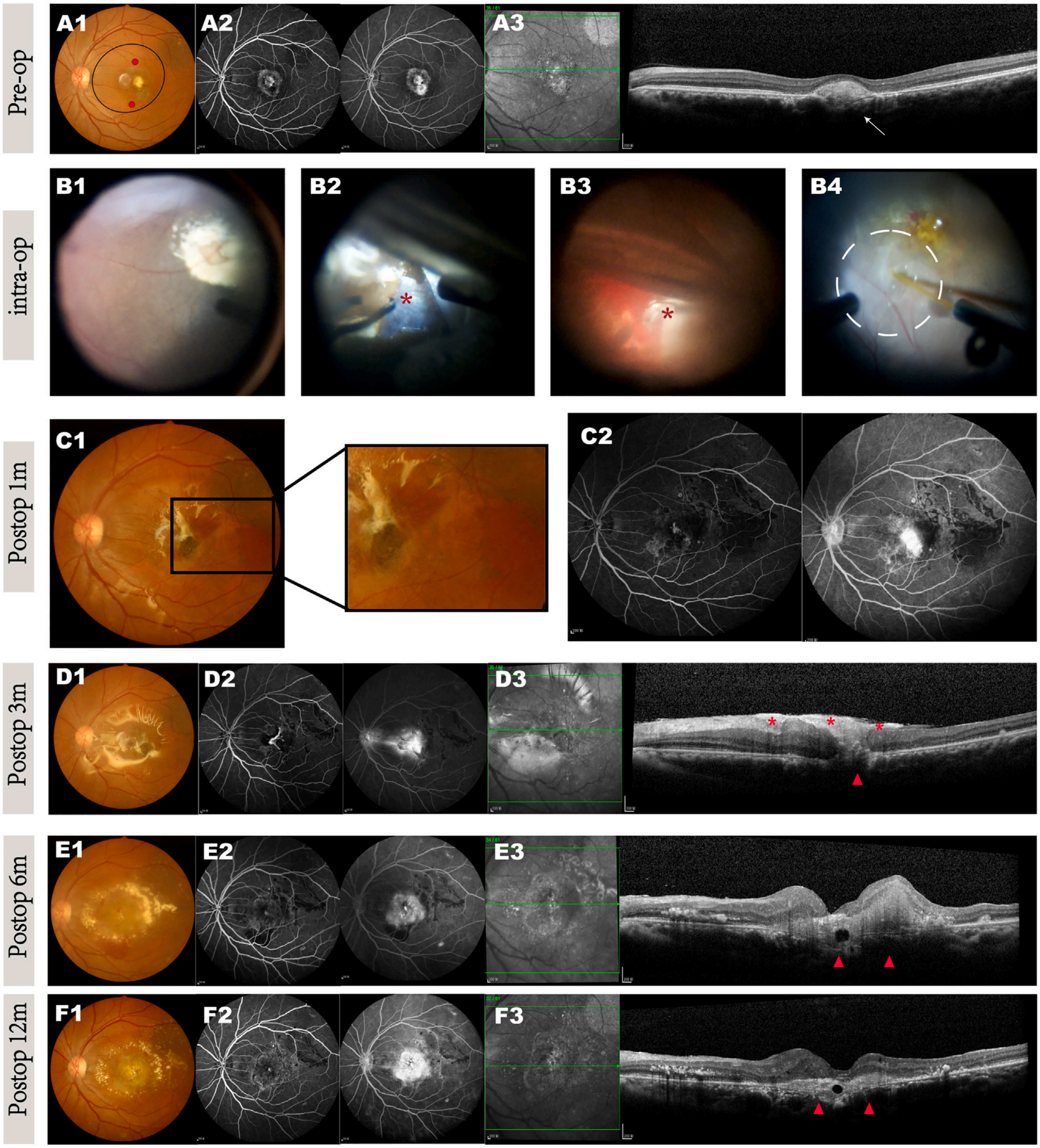

Figure 5. Retinal morphological change in the operated eye of patient 10

Retinal examination before and after CNV removal coupled with hESC-derived RPE suspension transplantation; preoperative (A1–A3), intraoperative (B1–B4), and postoperative images at 1 month (C1 and C2), 3 months (D1 and D3), 6 months (E1–E3), and 12 months (F1 and F3) are shown.

(A) Preop (A1–A3): (A1) fundus photography showing the fibrotic membrane in the macular area. The injection site is marked by red dots, and the formed subretinal bleb is indicated by a black circle. (A2) FFA images highlighting the typical CNV shapes without obvious late fluorescence leakage. (A3) SD-OCT scan shows fibrotic neovascular membrane breaks (arrow) under the RPE layer.

(legend continued on next page)

patient without CNV recurrence was treated with IVTA, and this therapy was highly effective. The patient with CNV recurrence was administered IVTA and anti-VEGF (ranibizumab) therapy, and the condition was well controlled (Brown et al., 2009). Although the anatomic recovery was not as good as that in group 1, the anti-inflammatory treatments reduced macular edema and fluorescence leakage in the FFA and prevented significant vision loss. CNV recurrence was also controlled with anti-VEGF treatment. This indicated that local immune rejection and CNV recurrence were controllable, and the transplantation of hESC-RPE cells combined with CNV removal was safe.

In our previous clinical trials of RPE transplantation for Stargardt macular degeneration (Li et al., 2021), we used a suspension of 1×10^5 RPE cells for transplantation. This low cell count was sufficient because the procedure targeted only the macular edge, which required a relatively small number of cells. However, in the present study, CNV removal resulted in a substantial loss of the RPE layer in the macular area, necessitating a significantly large number of RPE cells to adequately restore the damaged layer. Given the extremely low cell survival rates (less than 10%) observed in preclinical studies (Klassen et al., 2012; West et al., 2012), the dose of transplanted cells was increased by an order of magnitude. In a previous study, we transplanted 1×10^6 retinal progenitor cell suspensions into the macula of patients with retinitis pigmentosa. These findings confirmed the safety and tolerability of 1×10^6 cell transplantations in the macular area (Liu et al., 2017). In addition, previous studies have suggested that the standard dose for RPE transplantation in rats is approximately 1×10^5 cells/injection (Liu et al., 2018, 2023; Zhu et al., 2022). Considering the size difference between the rat retina (~80 mm^2) and the human retina (~10 cm^2) (Mayhew and Astle, 1997), we propose that an appropriate cell count for human RPE transplantation is ~1×10^6 RPE cells per eye.

Our results demonstrate that safe removal of CNV tissue without hemorrhage is a critical factor for successful transplantation of RPE cells in wet AMD. CNV removal creates a more stable microenvironment for the survival and integration of the transplanted RPE cells. Close adhesion of the CNV to the underlying tissue, as detected using OCT preoperatively, is a significant surgical risk factor. Patients with close CNV adhesions should be carefully evaluated, and alternative treatments may be more appropriate in such cases. Intraoperative hemorrhage resulting from choroid injury can adversely affect prognosis by increasing the risk of immune rejection and CNV recurrence due to severe breakdown of the BRB and the presence of residual CNV tissue. Although some patients in this study experienced hemorrhage during surgery and subsequently developed inflammation and CNV recurrence, these complications were effectively managed with appropriate interventions. Despite the theoretical increase in surgical risk, severe complications, such as RD and severe PVR, were not observed in our study. In contrast, the SST reported RD rates of 6% and 16%, with approximately half of these patients developing PVR (Bressler et al., 2004; Hawkins et al., 2004). Notably, all patients in our study remained stable or improved postoperatively, highlighting the reliability and potential benefits of this treatment approach.

In conclusion, our results suggest that surgical CNV removal coupled with hESC-derived RPE cell transplantation is a potentially safe treatment for wet AMD. We also report the factors contributing to unsatisfactory outcomes and share our experiences in managing complications in this study, which could help inform the preoperative screening of participants and postoperative management in future studies.

Limitations of the study

A limitation of this study is that patients needed to take immunosuppressants for 4 months, which is consistent with the findings of Schwartz et al. (2012). Although hESC-RPE

(B) The signal beneath the CNV was hyper-transmission. Intraop (B1–B4): (B1) intraoperative image before incising the peripheral temporal quadrant retina. (B2) After incising the retina, the fibrous membrane was tightly adhered to the underlying tissue. (B3) Significant fundus hemorrhage and residual lesion (asterisk) were observed due to the tight adhesion after complete removal of the CNV membrane. (B4) A subretinal bleb (marked by a dotted circle) formed after subretinal injection of transplanted cells. A small amount of subretinal blood remained visible in the macula.

(C) Postop 1 month (C1 and C2): (C1) blood is still present at the original lesion site on fundus photography. (C2) Late-phase fluorescence leakage at the macula on FFA.

(D) Postop 3 months (D1–D3): (D1) the subretinal hemorrhage has been fully absorbed. (D2) Increased fluorescence leakage in the late phase. (D3) Retinal edema persisted with ERM (asterisk).

(E) Postop 6 months after ERM peeling (E1–E3): (E1) spontaneous subretinal hemorrhage was observed beneath the macula, accompanied by a significant amount of exudate surrounding the macula. (E2) Persistent fluorescence leakage. (E3) Retinal edema did not improve, and intraretinal deposits were observed. Postoperative images taken 12 months after 3 consecutive anti-VEGF treatments.

(F) Postop 12 months (F1–F3): (F1) spontaneous bleeding has subsided, and the exudate remains. (F2) Fluorescence staining is visible in the late phase. (F3) The retina has flattened, and the edema has significantly subsided.

See also Figure S6.

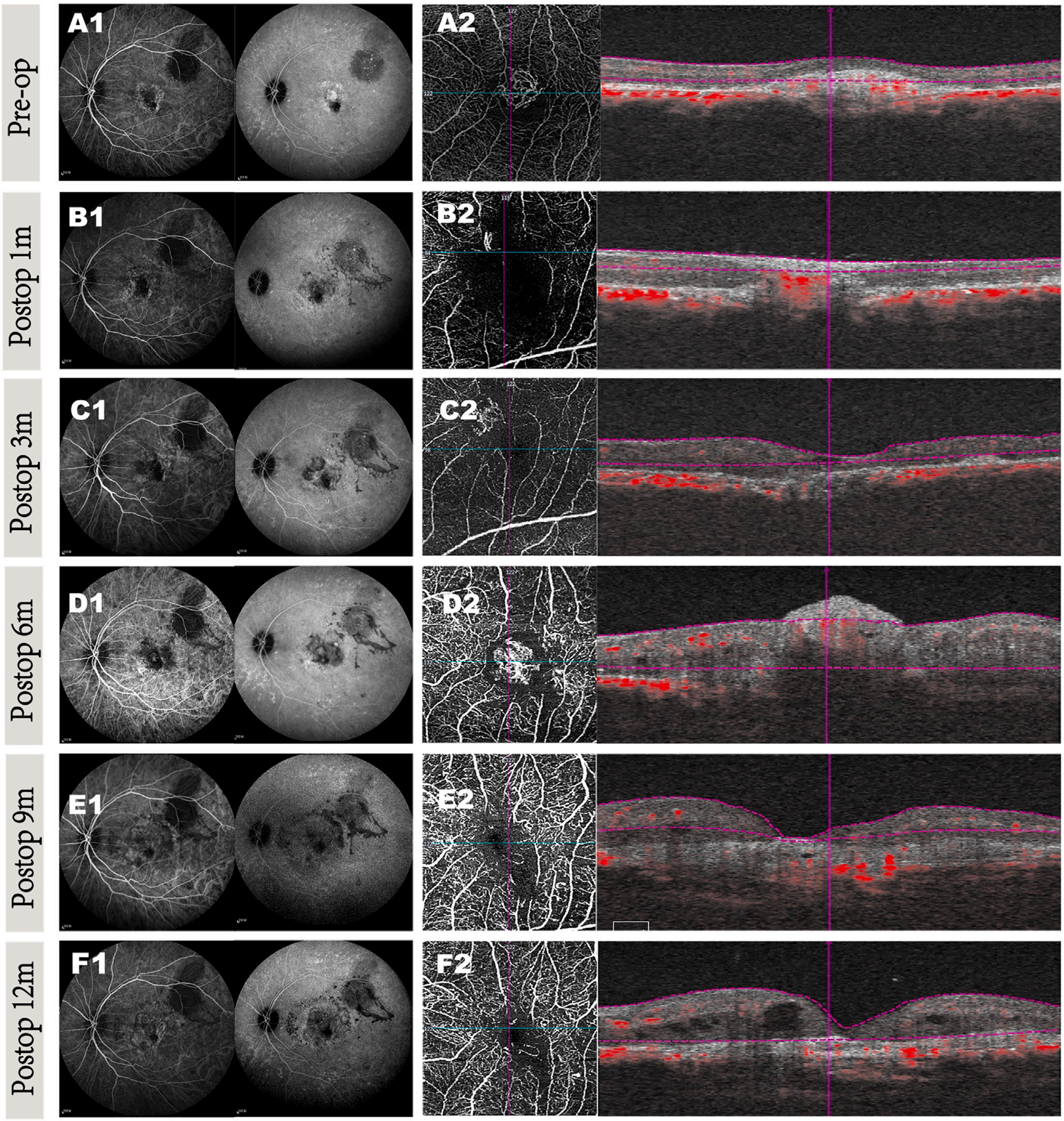

Figure 6. CNV changes before and after surgery over a 12-month period in patient 10

(A) Preop (A1 and A2): (A1) early (left side) and late (right side) phase of ICGA imaging. (A2) OCTA images revealing significant choroidal neovascularization blood signal in the macular area preoperatively.

(B–F) Postoperative images at 1 month (B1 and B2), 3 months (C1 and C2), 6 months (D1 and D2), 9 months (E1 and E2), and 12 months (F1 and F2): (B2) 1 month after surgery, the ICGA images show residual blood signal located at the original lesion site that has gradually developed into a new vessel network. OCTA scan suggests progressive retinal edema (C2–D2). After 3 consecutive intravitreal ranibizumab injections, the recurrent CNV was significantly reduced, leading to decreased blood flow signals beneath the retina and a noticeable reduction in retinal edema (E2–F2).

cells are considered hypoimmunogenic (Idelson et al., 2018), the risk of chronic immune rejection still exists. The strategy for immunosuppressive use in elderly patients after allograft cell transplantation remains inconclusive, and long-term immunosuppressant use may pose a higher risk of severe infections and cancer.

Compared with other embryonic stem cell-derived cell transplantation therapies, such as pancreas islet β cell transplantation for type 1 diabetes, hESC-RPE cells can be engineered using genome-editing techniques to allow escape from alloimmune and autoimmune reactions in the recipient (Loretelli et al., 2020). The human leukocyte antigen class I and II profiles of hESCs can be modified *in vitro*, and they can be induced to overexpress immunoregulatory factors such as PD-L1 and CD-47 (Han et al., 2019). These modified hESC-RPE cells would be invisible to both T and natural killer cell responses in all patients. This strategy could provide an off-the-shelf universal cell product that would avoid acute rejection and a chronic inflammatory response, and the patient would not require immunosuppression.

METHODS

Study design and participants

This phase 1, open-label, investigator-initiated trial enrolled 10 adult participants who met the inclusion and exclusion criteria. All patients provided written informed consent, and the trial was approved by the Ethics Committee of Southwest Hospital. The study was registered at ClinicalTrials.gov (NCT02749734).

Patients with binocular wet AMD were selected for intervention in the poorer-seeing eye (according to dominance and visual acuity) and were followed up for at least 12 months. Q-CTS-hESC-2 cells were obtained from the National Stem Cell Resource Center of China and verified by the Chinese National Institutes for Food and Drug Control (report number SH201402035) (Gu et al., 2017). The differentiation of Q-CTS-hESC-2-RPE cells followed established protocols to ensure safety and confirm RPE-specific properties prior to transplantation (Wu et al., 2016). After differentiation until passage 3 (120 days), the hESC-RPE cells exhibited typical cobblestone-like morphological characteristics and pigmentation. Biosafety analyses including fungal, bacterial, and viral examinations confirmed that the cells were free of microbial contaminants and endotoxins (Table S1). Immunostaining verified the proper differentiation of hESC-RPE cells, based on positive expression of RPE markers such as MITF, ZO-1, Bestrophin-1, REP-65, and CRALBP. Quantitative PCR further demonstrated high expression levels of RPE-related genes, including MITF, Bestrophin-1, REP-65, and CRALBP. Flow cytometry (fluorescence-activated cell sorting) analysis confirmed that the purity of the hESC-RPE cell cultures exceeded 99% for $\geq$1 RPE marker. Tumorigenicity tests were conducted on severe combined immunodeficiency mice, including a positive control group injected with undifferentiated hESCs. Subcutaneous injections of Q-CTS-hESC-2-RPE cells showed no signs of hyperproliferation or abnormal growth, whereas undifferentiated Q-CTS-hESC-2 cells developed into teratomas (Figure S1). These findings demonstrated that the hESC-RPE cells are suitable for clinical use.

Surgical procedure

The procedure involved 3-port pars plana vitrectomy with complete posterior vitreous detachment and total vitreous excision. RD in the superior temporal retina was achieved by injecting a balanced saline solution into the subretinal space, followed by peripheral retinotomy in the superior temporal quadrant. The subretinal CNV membrane was then exposed and carefully excised. Perfluorodecalin was applied to reattach the retina, and photocoagulation was used to seal the retinal breaks. Silicon oil tamponade was used, followed by an infusion of 100 μL of hESC-RPE cell suspension (containing 10^6 hESC-RPE cells) into the macular region using a 39-gauge cannula, creating a dome-shaped RD (Video S1). After surgery, patients were advised to remain in a supine position overnight until the subretinal fluid was absorbed, followed by a face-down position for 1 week.

Postoperative management

The patients were prescribed an oral immunosuppressive regimen based on Schwartz et al. (2012) with minor modifications (Liu et al., 2018). Silicone oil removal surgery was performed during the third month of follow-up.

Clinical evaluation

Patients were followed up periodically with comprehensive ophthalmic examinations, including BCVA using the ETDRS test, slit-lamp bio-microscopy, fundus examination, SD-OCT, FFA, ICGA, mfERG, microperimetry, and OCTA. Systemic health was monitored through physical examinations, vital sign assessments, electrocardiography, chest radiography, and hematological testing.

FFA and ICGA

A solution of sodium fluorescein (Alcon Laboratories, Inc., USA) and indocyanine green (Dandong Yichuang Pharmaceutical Co., Ltd, China) was injected intravenously. A confocal laser scanning system (Heidelberg Retina Angiography II, HRII, Heidelberg, Germany) was used to capture FFA and ICGA fundus images at the early, mid, and late time points.

SD-OCT

A Heidelberg SPECTRALIS HRA + OCT device (Heidelberg Engineering, Germany) was used, and all SD-OCT scans were obtained by the same experienced operator. Images were generated using a high-speed volumetric raster scan pattern over a 30° × 25° area. Each raster scan consisted of 61 horizontal line scans spaced 127 μm apart.

OCTA

An OCTA device (Carl Zeiss Meditec, Jena, Germany) was used, and 6 × 6-mm images were centered on the fovea (512 A-scans × 512 B-scans) of the participants. For each OCTA 6 × 6-mm volume, the inner retinal vascular networks, foveal avascular zone, and choriocapillaris were evaluated.

Immunosuppression protocol

Immunosuppressive drugs were administered according to the protocol described by Schwartz et al. (2012), with minor modifications. One week postoperatively, tacrolimus (serum concentration, 10 ng/mL), mycophenolate mofetil (200 mg, twice daily), and prednisone (30 mg, once daily) were administered and continued for 1 month. One month post-transplant, tacrolimus was slowly reduced to a serum level of 3–7 ng/mL. By 2 months post-transplant, prednisone was decreased to 15 mg/day, and mycophenolate mofetil was discontinued. Prednisone was discontinued 3 months post-transplant, and tacrolimus was reduced to a serum level of 3 ng/mL. All immunosuppressive drugs were discontinued 4 months post-transplant.

RESOURCE AVAILABILITY

Lead contact

Further information and requests for resources should be directed to and will be fulfilled by the corresponding author, Yong Liu (liuyong@tmmu.edu.cn).

Materials availability

This study did not generate new unique reagents.

Data and code availability

There are no accession numbers or genetic information relevant to this study.

ACKNOWLEDGMENTS

The authors thank the State Key Laboratory of Stem Cell and Reproductive Biology, Institute of Zoology, Chinese Academy of Sciences, for providing the embryonic stem cell sources. This work was supported by the National Key Research and Development Program of China (2024YFA1108705 to Prof. Y.L.), National Natural Science Foundation of China (82271132 and 81970843 to Prof. Y.L.), and Joint Logistic Support Force (no. 145BHQ090 00300X05).

AUTHOR CONTRIBUTIONS

Conceptualization and methodology: Y.L. and Z.Q.Y.; investigation: Y.X.L., S.Y.L., P.D., M.F.Z., B.L., Q.Y.L., and Y.L.; project administration and supervision: H.W., T.T.Z., and Y.L.; unrestricted access to all data: Y.X.L., H.W., and Y.L.; visualization: Y.X.L. and Q.Y.L.; writing – original draft: Y.X.L., H.W., and Y.L.; writing – review and editing: Y.X.L., H.W., T.T.Z., and Y.L.; funding acquisition: Y.L. All authors agreed to submit the manuscript, read and approved the final draft, and take full responsibility of its content, including the accuracy of the data and the fidelity of the trial to the registered protocol.

DECLARATION OF INTERESTS

All authors declare no competing interests.

SUPPLEMENTAL INFORMATION

Supplemental information can be found online at https://doi.org/10.1016/j.stemcr.2025.102424.

Received: September 14, 2024
Revised: January 28, 2025
Accepted: January 29, 2025
Published: February 27, 2025

REFERENCES

Alexander, P., Thomson, H.A.J., Luff, A.J., and Lotery, A.J. (2015). Retinal pigment epithelium transplantation: concepts, challenges, and future prospects. Eye *29*, 992–1002. https://doi.org/10.1038/eye.2015.89.

Bressler, N.M., Bressler, S.B., Childs, A.L., Haller, J.A., Hawkins, B.S., Lewis, H., MacCumber, M.W., Marsh, M.J., Redford, M., Sternberg, P., Jr., et al. (2004). Surgery for hemorrhagic choroidal neovascular lesions of age-related macular degeneration: ophthalmic findings: SST report no. 13. Ophthalmology *111*, 1993–2006. https://doi.org/10.1016/j.ophtha.2004.07.023.

Brown, D.M., Michels, M., Kaiser, P.K., Heier, J.S., Sy, J.P., and Ianchulev, T.; ANCHOR Study Group (2009). Ranibizumab versus verteporfin photodynamic therapy for neovascular age-related macular degeneration: Two-year results of the ANCHOR study. Ophthalmology *116*, 57–65.e5. https://doi.org/10.1016/j.ophtha.2008.10.018.

da Cruz, L., Fynes, K., Georgiadis, O., Kerby, J., Luo, Y.H., Ahmado, A., Vernon, A., Daniels, J.T., Nommiste, B., Hasan, S.M., et al. (2018). Phase 1 clinical study of an embryonic stem cell-derived retinal pigment epithelium patch in age-related macular degeneration. Nat. Biotechnol. *36*, 328–337. https://doi.org/10.1038/nbt.4114.

Deng, Y., Qiao, L., Du, M., Qu, C., Wan, L., Li, J., and Huang, L. (2022). Age-related macular degeneration: Epidemiology, genetics, pathophysiology, diagnosis, and targeted therapy. Genes Dis. *9*, 62–79. https://doi.org/10.1016/j.gendis.2021.02.009.

Diniz, B., Thomas, P., Thomas, B., Ribeiro, R., Hu, Y., Brant, R., Ahuja, A., Zhu, D., Liu, L., Koss, M., et al. (2013). Subretinal implantation of retinal pigment epithelial cells derived from human

embryonic stem cells: improved survival when implanted as a monolayer. Investig. Ophthalmol. Vis. Sci. *54*, 5087–5096. https://doi.org/10.1167/iovs.12-11239.

Falkner, C.I., Leitich, H., Frommlet, F., Bauer, P., and Binder, S. (2007). The end of submacular surgery for age-related macular degeneration? A meta-analysis. Graefes Arch. Clin. Exp. Ophthalmol. *245*, 490–501. https://doi.org/10.1007/s00417-005-0184-3.

Friedman, D.S., O'Colmain, B.J., Muñoz, B., Tomany, S.C., McCarty, C., de Jong, P.T.V.M., Nemesure, B., Mitchell, P., and Kempen, J.; Eye Diseases Prevalence Research Group (2004). Prevalence of age-related macular degeneration in the United States. Arch. Ophthalmol. *122*, 564–572. https://doi.org/10.1001/archopht.122.4.564.

Grossniklaus, H.E., and Gass, J.D. (1998). Clinicopathologic correlations of surgically excised type 1 and type 2 submacular choroidal neovascular membranes. Am. J. Ophthalmol. *126*, 59–69. https://doi.org/10.1016/s0002-9394(98)00145-7.

Gu, Q., Wang, J., Wang, L., Liu, Z.X., Zhu, W.W., Tan, Y.Q., Han, W.F., Wu, J., Feng, C.J., Fang, J.H., et al. (2017). Accreditation of Biosafe Clinical-Grade Human Embryonic Stem Cells According to Chinese Regulations. Stem Cell Rep. *9*, 366–380. https://doi.org/10.1016/j.stemcr.2017.04.017.

Guthoff, R., and Schrader, W. (2004). Longterm results in surgical removal of subfoveal choroidal neovascularization in age-related macular degeneration. Acta Ophthalmol. Scand. *82*, 686–690. https://doi.org/10.1111/j.1600-0420.2004.00338.x.

Han, X., Wang, M., Duan, S., Franco, P.J., Kenty, J.H.R., Hedrick, P., Xia, Y., Allen, A., Ferreira, L.M.R., Strominger, J.L., et al. (2019). Generation of hypoimmunogenic human pluripotent stem cells. Proc. Natl. Acad. Sci. USA *116*, 10441–10446. https://doi.org/10.1073/pnas.1902566116.

Hawkins, B.S., Bressler, N.M., Miskala, P.H., Bressler, S.B., Holekamp, N.M., Marsh, M.J., Redford, M., Schwartz, S.D., Sternberg, P., Jr., Thomas, M.A., et al. (2004). Surgery for subfoveal choroidal neovascularization in age-related macular degeneration: ophthalmic findings: SST report no. 11. Ophthalmology *111*, 1967–1980. https://doi.org/10.1016/j.ophtha.2004.07.021.

Idelson, M., Alper, R., Obolensky, A., Yachimovich-Cohen, N., Rachmilewitz, J., Ejzenberg, A., Beider, E., Banin, E., and Reubinoff, B. (2018). Immunological Properties of Human Embryonic Stem Cell-Derived Retinal Pigment Epithelial Cells. Stem Cell Rep. *11*, 681–695. https://doi.org/10.1016/j.stemcr.2018.07.009.

Kashani, A.H., Lebkowski, J.S., Rahhal, F.M., Avery, R.L., Salehi-Had, H., Dang, W., Lin, C.M., Mitra, D., Zhu, D., Thomas, B.B., et al. (2018). A bioengineered retinal pigment epithelial monolayer for advanced, dry age-related macular degeneration. Sci. Transl. Med. *10*, eaao4097. https://doi.org/10.1126/scitranslmed.aao4097.

Klassen, H., Kiilgaard, J.F., Warfvinge, K., Samuel, M.S., Prather, R.S., Wong, F., Petters, R.M., la Cour, M., and Young, M.J. (2012). Photoreceptor Differentiation following Transplantation of Allogeneic Retinal Progenitor Cells to the Dystrophic Rhodopsin Pro347Leu Transgenic Pig. Stem Cell. Int. *2012*, 939801. https://doi.org/10.1155/2012/939801.

Li, S.Y., Liu, Y., Wang, L., Wang, F., Zhao, T.T., Li, Q.Y., Xu, H.W., Meng, X.H., Hao, J., Zhou, Q., et al. (2021). A phase I clinical trial of human embryonic stem cell-derived retinal pigment epithelial cells for early-stage Stargardt macular degeneration: 5-years' follow-up. Cell Prolif. *54*, e13100. https://doi.org/10.1111/cpr.13100.

Liu, Q., Liu, J., Guo, M., Sung, T.C., Wang, T., Yu, T., Tian, Z., Fan, G., Wu, W., and Higuchi, A. (2023). Comparison of retinal degeneration treatment with four types of different mesenchymal stem cells, human induced pluripotent stem cells and RPE cells in a rat retinal degeneration model. J. Transl. Med. *21*, 910. https://doi.org/10.1186/s12967-023-04785-1.

Liu, Y., Chen, S.J., Li, S.Y., Qu, L.H., Meng, X.H., Wang, Y., Xu, H.W., Liang, Z.Q., and Yin, Z.Q. (2017). Long-term safety of human retinal progenitor cell transplantation in retinitis pigmentosa patients. Stem Cell Res. Ther. *8*, 209. https://doi.org/10.1186/s13287-017-0661-8.

Liu, Y., Xu, H.W., Wang, L., Li, S.Y., Zhao, C.J., Hao, J., Li, Q.Y., Zhao, T.T., Wu, W., Wang, Y., et al. (2018). Human embryonic stem cell-derived retinal pigment epithelium transplants as a potential treatment for wet age-related macular degeneration. Cell Discov. *4*, 50. https://doi.org/10.1038/s41421-018-0053-y.

Loretelli, C., Assi, E., Seelam, A.J., Ben Nasr, M., and Fiorina, P. (2020). Cell therapy for type 1 diabetes. Expet Opin. Biol. Ther. *20*, 887–897. https://doi.org/10.1080/14712598.2020.1748596.

Lu, B., Malcuit, C., Wang, S., Girman, S., Francis, P., Lemieux, L., Lanza, R., and Lund, R. (2009). Long-term safety and function of RPE from human embryonic stem cells in preclinical models of macular degeneration. Stem Cell. *27*, 2126–2135. https://doi.org/10.1002/stem.149.

Mandai, M., Watanabe, A., Kurimoto, Y., Hirami, Y., Morinaga, C., Daimon, T., Fujihara, M., Akimaru, H., Sakai, N., Shibata, Y., et al. (2017). Autologous Induced Stem-Cell-Derived Retinal Cells for Macular Degeneration. N. Engl. J. Med. *376*, 1038–1046. https://doi.org/10.1056/NEJMoa1608368.

Mayhew, T.M., and Astle, D. (1997). Photoreceptor number and outer segment disk membrane surface area in the retina of the rat: stereological data for whole organ and average photoreceptor cell. J. Neurocytol. *26*, 53–61. https://doi.org/10.1023/a:1018563409196.

Mehat, M.S., Sundaram, V., Ripamonti, C., Robson, A.G., Smith, A.J., Borooah, S., Robinson, M., Rosenthal, A.N., Innes, W., Weleber, R.G., et al. (2018). Transplantation of Human Embryonic Stem Cell-Derived Retinal Pigment Epithelial Cells in Macular Degeneration. Ophthalmology *125*, 1765–1775. https://doi.org/10.1016/j.ophtha.2018.04.037.

Merrill, P.T., LoRusso, F.J., Lomeo, M.D., Saxe, S.J., Khan, M.M., and Lambert, H.M. (1999). Surgical removal of subfoveal choroidal neovascularization in age-related macular degeneration. Ophthalmology *106*, 782–789. https://doi.org/10.1016/s0161-6420(99)90167-7.

Schwartz, S.D., Hubschman, J.P., Heilwell, G., Franco-Cardenas, V., Pan, C.K., Ostrick, R.M., Mickunas, E., Gay, R., Klimanskaya, I., and Lanza, R. (2012). Embryonic stem cell trials for macular degeneration: a preliminary report. Lancet *379*, 713–720. https://doi.org/10.1016/s0140-6736(12)60028-2.

Schwartz, S.D., Regillo, C.D., Lam, B.L., Eliott, D., Rosenfeld, P.J., Gregori, N.Z., Hubschman, J.P., Davis, J.L., Heilwell, G., Spirn, M., et al. (2015). Human embryonic stem cell-derived retinal pigment epithelium in patients with age-related macular degeneration and Stargardt's macular dystrophy: follow-up of two open-label phase 1/2 studies. Lancet *385*, 509–516. https://doi.org/10.1016/s0140-6736(14)61376-3.

Song, W.K., Park, K.M., Kim, H.J., Lee, J.H., Choi, J., Chong, S.Y., Shim, S.H., Del Priore, L.V., and Lanza, R. (2015). Treatment of macular degeneration using embryonic stem cell-derived retinal pigment epithelium: preliminary results in Asian patients. Stem Cell Rep. *4*, 860–872. https://doi.org/10.1016/j.stemcr.2015.04.005.

Spraul, C.W., Lang, G.E., Grossniklaus, H.E., and Lang, G.K. (1999). Histologic and morphometric analysis of the choroid, Bruch's membrane, and retinal pigment epithelium in postmortem eyes with age-related macular degeneration and histologic examination of surgically excised choroidal neovascular membranes. Surv. Ophthalmol. *44*, S10–S32. https://doi.org/10.1016/s0039-6257(99)00086-7.

Sugita, S., Mandai, M., Hirami, Y., Takagi, S., Maeda, T., Fujihara, M., Matsuzaki, M., Yamamoto, M., Iseki, K., Hayashi, N., et al. (2020). HLA-Matched Allogeneic iPS Cells-Derived RPE Transplantation for Macular Degeneration. J. Clin. Med. *9*, 2217. https://doi.org/10.3390/jcm9072217.

Takagi, S., Mandai, M., Gocho, K., Hirami, Y., Yamamoto, M., Fujihara, M., Sugita, S., Kurimoto, Y., and Takahashi, M. (2019). Evaluation of Transplanted Autologous Induced Pluripotent Stem Cell-Derived Retinal Pigment Epithelium in Exudative Age-Related Macular Degeneration. Ophthalmol. Retina *3*, 850–859. https://doi.org/10.1016/j.oret.2019.04.021.

Thomas, M.A., Grand, M.G., Williams, D.F., Lee, C.M., Pesin, S.R., and Lowe, M.A. (1992). Surgical management of subfoveal choroidal neovascularization. Ophthalmology *99*, 952–976. discussion 975-956. https://doi.org/10.1016/s0161-6420(92)31888-3.

Uppal, G., Milliken, A., Lee, J., Acheson, J., Hykin, P., Tufail, A., and da Cruz, L. (2007). New algorithm for assessing patient suitability for macular translocation surgery. Clin. Exp. Ophthalmol. *35*, 448–457. https://doi.org/10.1111/j.1442-9071.2007.01528.x.

Wang, S., Lu, B., Girman, S., Holmes, T., Bischoff, N., and Lund, R.D. (2008). Morphological and functional rescue in RCS rats after RPE cell line transplantation at a later stage of degeneration. Investig. Ophthalmol. Vis. Sci. *49*, 416–421. https://doi.org/10.1167/iovs.07-0992.

West, E.L., Gonzalez-Cordero, A., Hippert, C., Osakada, F., Martinez-Barbera, J.P., Pearson, R.A., Sowden, J.C., Takahashi, M., and Ali, R.R. (2012). Defining the integration capacity of embryonic stem cell-derived photoreceptor precursors. Stem Cell. *30*, 1424–1435. https://doi.org/10.1002/stem.1123.

Wong, W.L., Su, X., Li, X., Cheung, C.M.G., Klein, R., Cheng, C.Y., and Wong, T.Y. (2014). Global prevalence of age-related macular degeneration and disease burden projection for 2020 and 2040: a systematic review and meta-analysis. Lancet Global Health *2*, e106–e116. https://doi.org/10.1016/s2214-109x(13)70145-1.

Wu, W., Zeng, Y., Li, Z., Li, Q., Xu, H., and Yin, Z.Q. (2016). Features specific to retinal pigment epithelium cells derived from three-dimensional human embryonic stem cell cultures - a new donor for cell therapy. Oncotarget *7*, 22819–22833. https://doi.org/10.18632/oncotarget.8185.

Zhou, R., and Caspi, R.R. (2010). Ocular immune privilege. F1000 Biol. Rep. *2*, 3. https://doi.org/10.3410/b2-3.

Zhu, X., Chen, Z., Wang, L., Ou, Q., Feng, Z., Xiao, H., Shen, Q., Li, Y., Jin, C., Xu, J.Y., et al. (2022). Direct conversion of human umbilical cord mesenchymal stem cells into retinal pigment epithelial cells for treatment of retinal degeneration. Cell Death Dis. *13*, 785. https://doi.org/10.1038/s41419-022-05199-5.

Stem Cell Reports, Volume *20*

Supplemental Information

Safe CNV removal is crucial for successful hESC-RPE transplantation in wet age-related macular degeneration

Ying Xue Lv, Qi You Li, Ping Duan, Min Fang Zhang, Bo Liu, Shi Ying Li, Tong Tao Zhao, Hao Wang, Yong Liu, and Zheng Qin Yin

Supplemental Information

Supplemental Figures

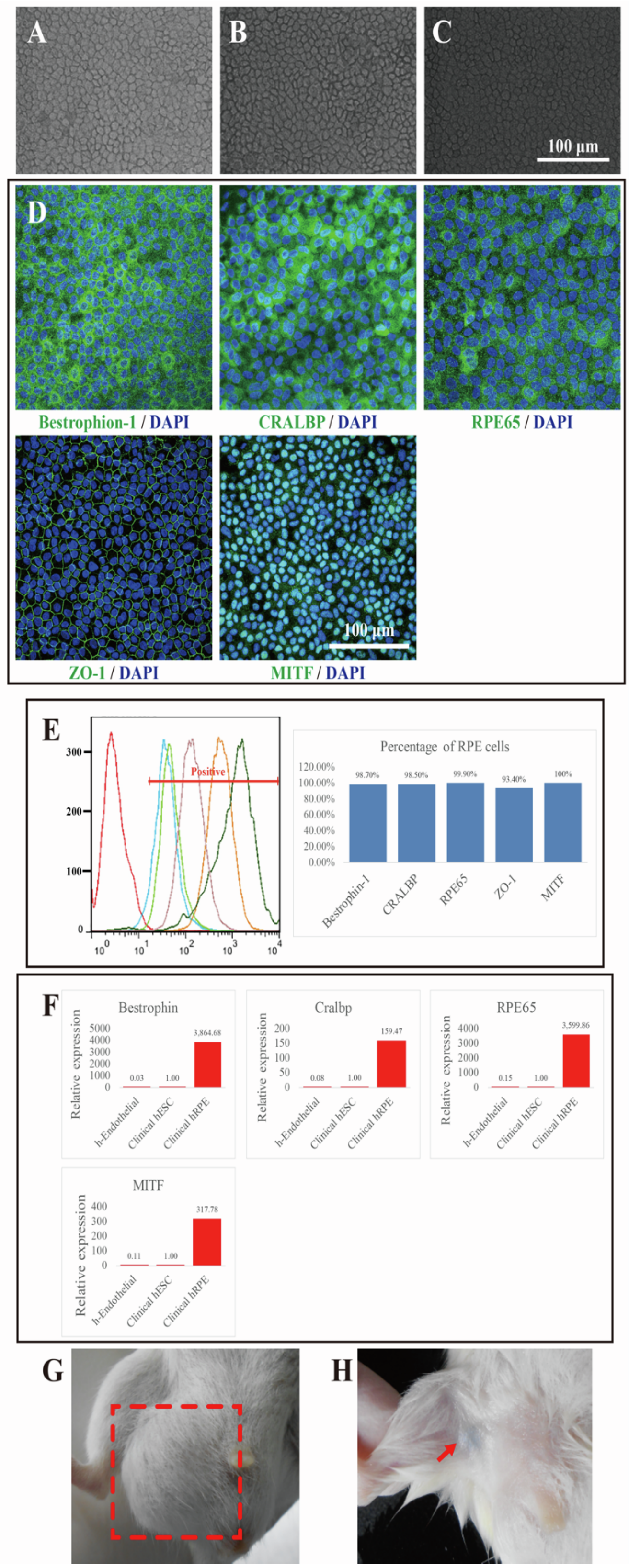

Figure S1. hESC-RPE cell identification of transplantation for patients. (A-C) Brightfield microphotography of induced hESC-RPE at passage 3 shows that the confluent cobblestone monolayer with pigment accumulation (A-patient 1, B-patient 2, C-patient 3). (D) Immunofluorescence staining assay. The tight junction complex protein (ZO-1), early neural and eye field markers (MITF), RPE-specific markers (Bestrophin-1, CRALBP, RPE65) (green) with corresponding DAPI (blue). (E) Flow cytometric analysis. (F) Quantitative PCR shows up-regulation of RPE cell-specific genes, including Bestrophin-1, CRALBP, RPE65 and MITF. (G-H) Tumorigenicity testing in SCID mice. Three months after subcutaneously injection, teratomas developed in mouse with Q-CTS-hESC-2 cells (G, red dotted box), and no hyperproliferation or abnormal growth in mouse with CTS-hESC2-RPE cells (H, red arrow).

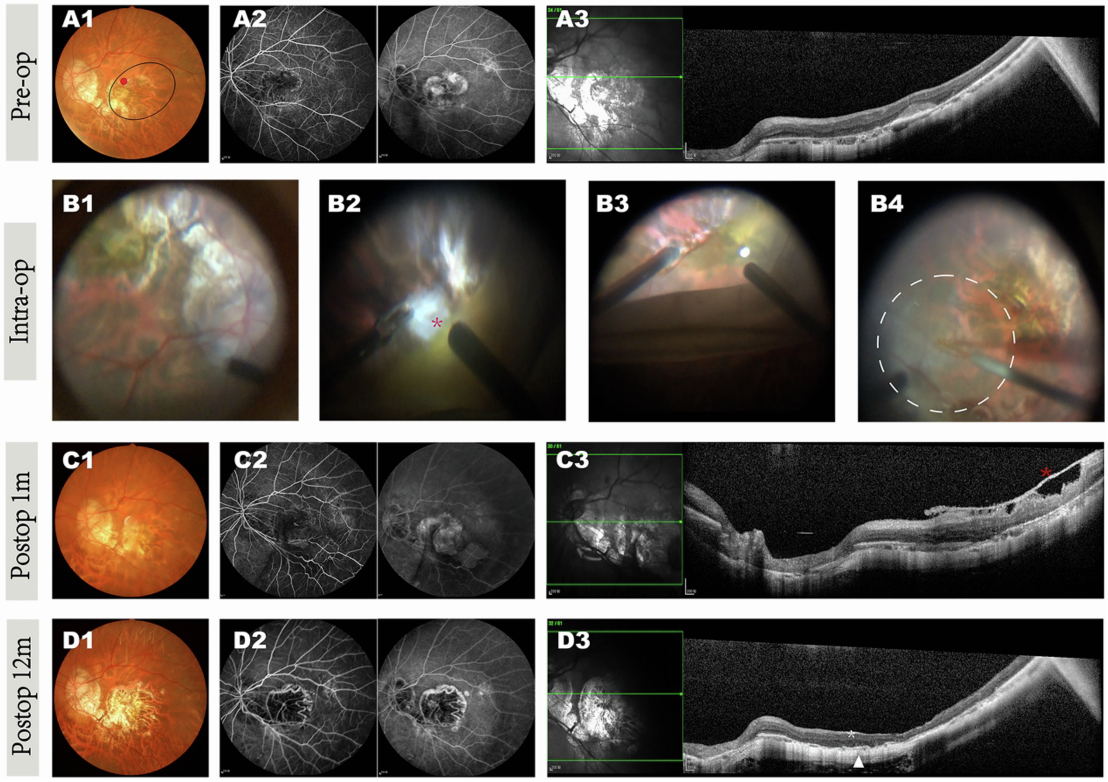

Figure S2. Retinal morphological changes in the operated eye of patient 2. Retinal examination before and after choroidal neovascularization (CNV) removal coupled with hESC-derived RPE suspension transplantation, at pre-operative (A1-A3), intra-operative (B1-B4), post-operative 1-month (C1-C3), and post-operative 12 months (D1-D3) stages. Pre-operative (A1-A3): (A1) Fundus photography showing the fibrotic membrane in the macular area. The injection site was marked by red dots and the formed subretinal bleb was indicated by a black circle. (A2) FFA images highlighting the typical shape of CNV. (A3) SD-OCT scan shows a hyperreflective mass broken through the RPE layer. The signal beneath the CNV was hypo-transmission. Intra-operative (B1-B4): (B1) Intraoperative image before incising the peripheral temporal quadrant retina. (B2) After incising the retina, the fiber membrane (red asterisk) is visible and easy to peel off without choroidal vessels damage. (B3) No fundus hemorrhage was observed after complete removal of the CNV membrane. (B4) A subretinal

bleb was formed after subretinal injection of transplanted cells, marked by a dashed circle. Post-operative 1-month (C1-C3): (C1) Fundus photo showing no subretinal pigmentation, hemorrhage or recurrent CNV, but macular geographic atrophy developed. (C2) The FFA shows no fluorescence leakage that suggestive of recurrent CNV network. (C3) SD-OCT image demonstrates no residual CNV lesion but development of ERM (asterisk) and retinal edema. Post-operative 6 months (D1-D3): (D1) FFA images reveal no subretinal pigmentation, hemorrhage or recurrent CNV, but macular geographic atrophy developed. (D2) The FFA shows no fluorescence leakage. (D3) SD-OCT image demonstrates a hyperreflective layer-like structure (arrowheads) over the bare Bruch’s membrane, with the relatively good restoration of ONL (white asterisk).

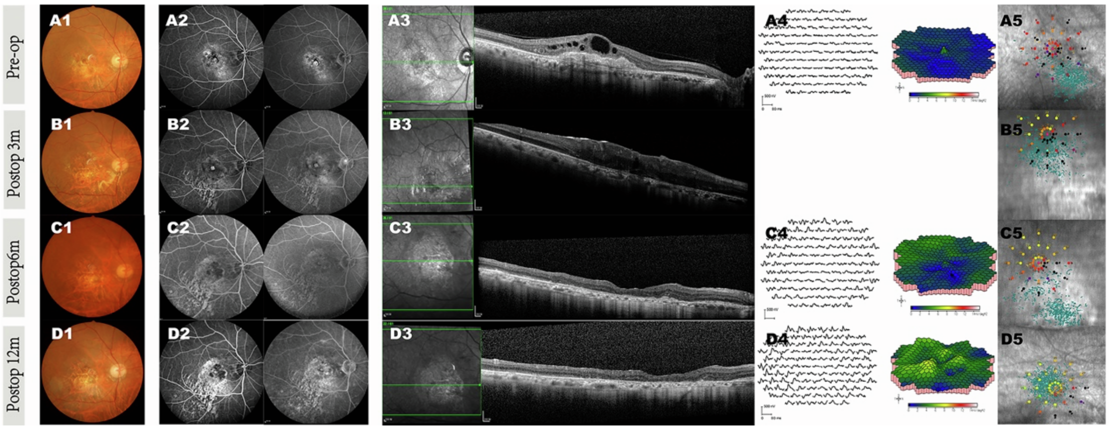

Figure S3. Retinal morphological and functional changes in the operated eye of patient 3. Fundus photography (1), FFA images (2), SD-OCT scan (3), mf-ERG (4) and microperimetry (5) at pre-operative (A), post-operative 3-month (B), post-operative 6-month (C), and post-operative 12 months (D) stages. Preoperatively, CNV lesion with cystoid macular edema were seen and FFA images indicated the leakage of the lesion. ERM occurred at 3 months and caused corresponding macular edema. After peeling the membrane, the retinal edema and the late fluorescence leakage subsided. No residual or recurrent CNV lesion was observed. Retinal function remained stable throughout the follow-up.

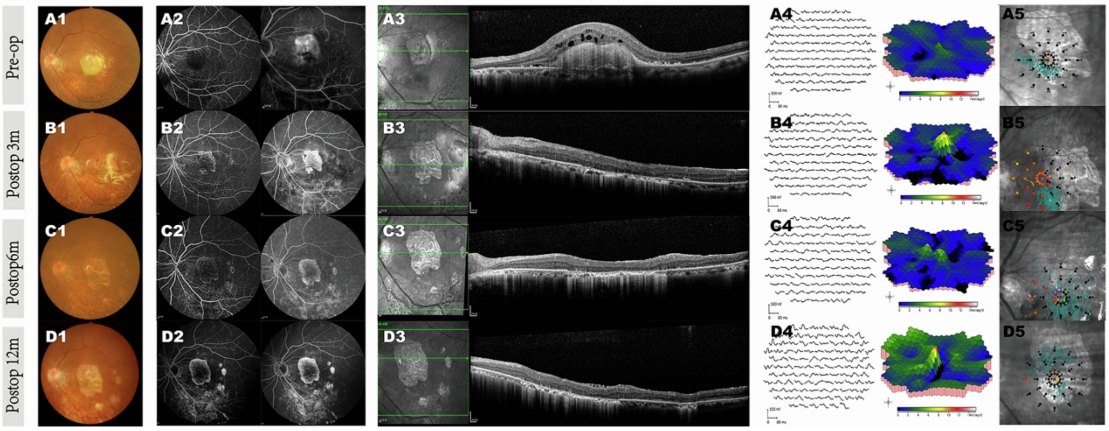

Figure S4. Retinal morphological and functional changes in the operated eye of patient 4. Fundus photography (1), FFA images (2), SD-OCT scan (3), mf-ERG (4) and microperimetry (5) at pre-operative (A), post-operative 3-month (B), post-operative 6-month (C), and post-operative 12 months (D) stages. Preoperatively, typical CNV lesion with retinal edema and late fluorescence leakage were presented. The retinal structure recovered with no recurrent edema or leakage postoperatively. No residual or recurrent CNV lesion was observed. Retinal function remained stable until the last visit.

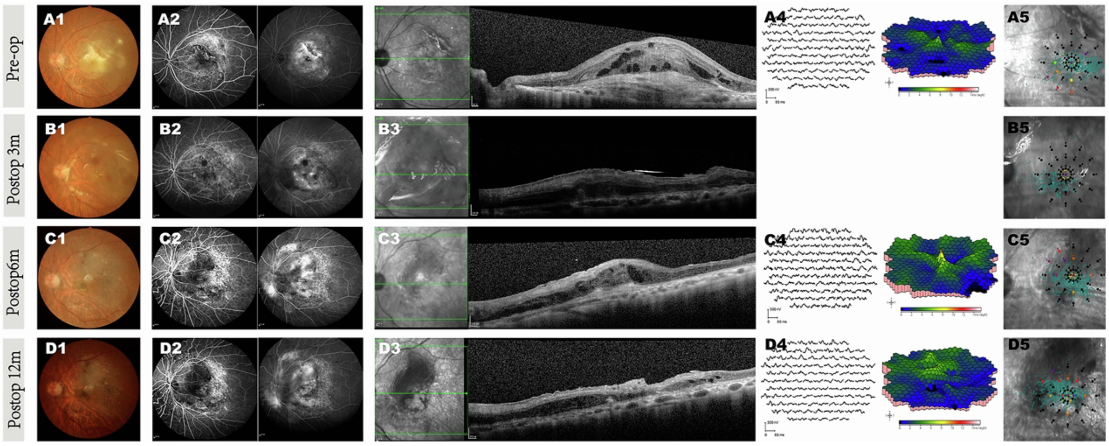

Figure S5. Retinal morphological and functional changes in the operated eye of patient 9. Fundus photography (1), FFA images (2), SD-OCT scan (3), mf-ERG (4) and microperimetry (5) at pre-operative (A), post-operative 3-month (B), post-operative 6-month (C), and post-operative 12 months (D) stages. Preoperatively, the fibrotic membrane and elevated retina were presented in the macular area. During surgery, hemorrhage occurred when removing the CNV. The retina edema persisted after ERM removal at 3 months postoperatively. After subsequent intravitreal TA injections, the retina became flatten and the fluorescence leakage subsided. No residual or recurrent CNV lesion were observed. Retina function remained stable throughout the follow-up.

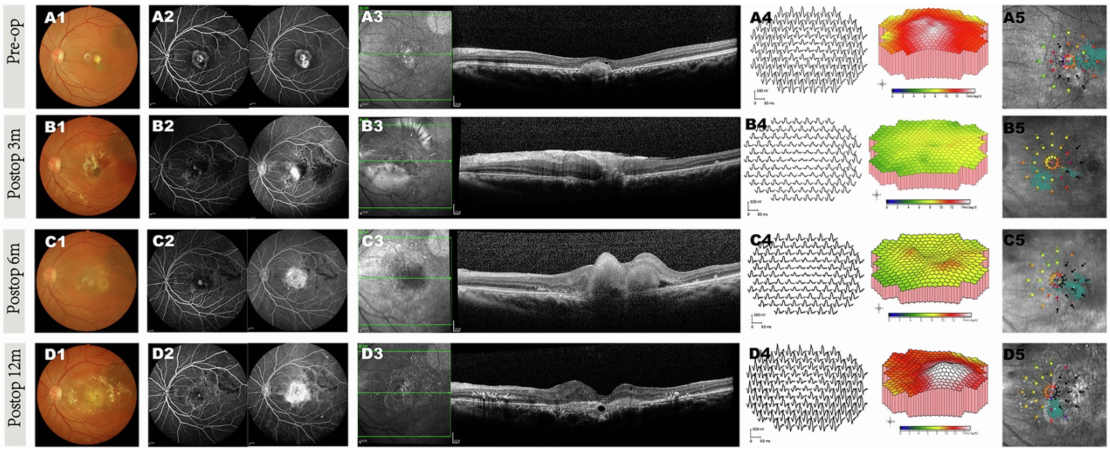

Figure S6. Retinal morphological and functional changes in the operated eye of patient 10. Fundus photography (1), FFA images (2), SD-OCT scan (3), mf-ERG (4) and microperimetry (5) at pre-operative (A), post-operative 3-month (B), post-operative 6-month (C), and post-operative 12 months (D) stages. Preoperatively, the fibrotic neovascular membrane under the RPE layer was visible, and FFA images showed the typical CNV shapes without obvious late fluorescence leakage. Spontaneous subretinal hemorrhage and increased fluorescence leakage in the late stage were observed. ERM peeling did not improve these presentations. However, after three consecutive anti-VEGF treatments, the retina became flattened, and the edema significantly subsided. The residual and recurrent CNV lesions were observed by OCTA. Retina function remained stable throughout the follow-up.

Supplemental Tables

Table S1. Q-CTS-hESC-2-RPE biosafety testing.

Test	Specification	Clinical hESC-RPE Cell
Fungus	Negative	Negative
Bacterium	Negative	Negative
Mycoplasma	Negative	Negative
Hepatitis B Virus (HBV)	Negative	Negative
Human Immunodeficiency Virus (HIV)	Negative	Negative
Hepatitis C Virus (HCV)	Negative	Negative
Treponema Pallidum (TP)	Negative	Negative
Endotoxin	<0.50EU/mL	<0.50EU/mL

Table S2. The sequences of the primers used to amplify hRPE cell specific genes.

Gene name	Forward primers	Reverse primers	Product size(bp)
MITF (NM_198159)	TTCACGAGCGTCCTGTATGCAGAT	AGTTTCCCGAGACAGGCAACGTAT	149
BESTROPHIN (NM_004183)	TATGACTCCGGCAGAACACAAGCA	TGTTCATCTCGTTCAGCAGGCTCT	161
RPE-65	TGCTTACGTACGGGCAATGACTGA	AGTTGGTCTCTGTGCAAGCGTAGT	191
CRALBP	TTCAAGGGCTTTACCATGCAGCAG	AGTACCATGGCTGGTGGATGAAGT	130
GAPDH (human)	agaaggctggggctcatttg	aggggccatccacagtcttc	

Supplemental Methods

hESC-RPE Cell Identification

Immunostaining

hESC-RPE cells grown on cover-slips were fixed with 4% paraformaldehyde for 15 min, permeabilized using 0.1% Triton X-100 in PBS for 15 min, and blocked for 60 min in 5% goat serum. Primary antibodies against MITF, ZO1, and RPE cell markers including Cralbp, Bestrophin-1 and RPE65 were diluted in the same blocking buffer and incubated with the samples overnight at 4°C, washed, and then incubated with a fluorescently coupled secondary antibody for 1 h at room temperature. The nuclei were stained with 4,6-diamidino-2-phenylindole (DAPI; Invitrogen). Fluorescent images were acquired with a confocal microscope (Zeiss LSM 700, Carl Zeiss; software: Zen lite 2012)[1].

Flow cytometry

Flow cytometry was used to identify the purity of hESC-RPE cells[2-5]. Briefly, cells cultured in growth medium or differentiation medium were detached using HyQTase (Thermo Fisher Scientific) and collected. Cells were blocked with CD32/16 (Bio- Legend) and then incubated for 30 min at 4°C with primary antibodies to surface markers (1:30) or an isotype control (1:30; BioLegend). Cells were rinsed with staining buffer (eBioscience) and a permeabilization buffer (eBioscience) after each step in the procedure. Cells were counted using a FACS Calibur Flow Cytometer and at least 10,000 events were collected for each sample; samples were analyzed using FlowJo software (FlowJo, Ashland, OR, USA).

Real-time PCR analysis

Total RNA was extracted from induced hESC-RPE cells (P3) using TRIzol reagent (9109, TAKARA, Japan) according to the manufacturer's protocol. The eluted RNA was dissolved in sterile DEPC water, and the total RNA concentration was then measured with a spectrophotometer. cDNA synthesis and qPCR following the protocol in the TAKARA user's manual (code no. RR047A). The sequences of the primers used to amplify hRPE cellspecific genes are shown in Supplementary Table S2.

Teratoma formation

100 μl of hESC or hESC- RPE cell suspension (1×10^7 cells) was subcutaneously implanted into the groin of SCID mice (regardless of sex)[4]. Mice were maintained under pathogen-free conditions at the animal facility of the Third Military Medical University and received humane care according to the criteria outlined by the requirements of the Laboratory Animal Welfare and Ethics Committee of the Third Military Medical University. We monitored the tumor size and condition of the mice weekly. The animals were anesthetized with overdose three months later and the tissue was examined by a pathologist to identify microscopic pathological changes and evidence of tumor formation.

Clinical Evaluation

mfERG

Multifocal electroretinography (mfERG) elicited by a Veris system (Electro-Diagnostic Imaging, Inc., Burlingame, CA, U.S.A.) was used to test the local function of the macula. All tests were performed according to the International Society for Clinical Electrophysiology of Vision (ISCVE) Standards and repeated three times in every test[6, 7]. The test protocols we used were as follows: the "M-sequence" algorithm was used to control the temporal sequence of change between the light and dark stages of each stimulus hexagon. The stimulus field contained 103 hexagons with a field diameter of 40°. The pupils were fully dilated, bipolar corneal contact electrodes attached, and a real-time fundus camera used to monitor eye movements during the test. Monocular stimulation was used for a four min recording session band-pass filtered at 10-300 Hz. The standard measurement for mfERG amplitude density is the trough-to-peak amplitude (nv) of the N1 and P1 responses over its hexagon in degrees (nv/deg2). Groups of responses from the central to the peripheral retina can be divided into rings 1 to 6.

Microperimetry

All patients underwent four times microperimetry tests before and after surgery[8]. The pupillary was dilated with 0.5% Tropicamide before examination. Exams were performed under dim-light conditions, while the fellow eye was patched. A grid of 45 spots was centered on the fovea

region. The stimulus was a Goldmann III white spot with a stimulus duration of 200 ms. The threshold strategy was HFA 4-2, and background luminance was 31.4 asb. Stimulus attenuation ranged from 0 dB to 34 dB. Total mean retinal sensitivity (MRS) (12° circle), 95 bivariate contour ellipse area (BCEA) (as the area of an ellipse that encompasses 95.4% of fixation points), MRS in the ellipse area, and percentage of fixation points in a circle of 2° (p1) and 4° (p2) diameter were recorded.

Supplemental References:

1. Gao, L., et al., *Intermittent high oxygen influences the formation of neural retinal tissue from human embryonic stem cells.* Sci Rep, 2016. **6**: p. 29944.
2. Osakada, F., et al., *Toward the generation of rod and cone photoreceptors from mouse, monkey and human embryonic stem cells.* Nat Biotechnol, 2008. **26**(2): p. 215-24.
3. Wu, W., et al., *Features specific to retinal pigment epithelium cells derived from three-dimensional human embryonic stem cell cultures - a new donor for cell therapy.* Oncotarget, 2016. **7**(16): p. 22819-33.
4. Li, Q.Y., et al., *Functional assessment of cryopreserved clinical grade hESC-RPE cells as a qualified cell source for stem cell therapy of retinal degenerative diseases.* Exp Eye Res, 2021. **202**: p. 108305.
5. Schwartz, S.D., et al., *Embryonic stem cell trials for macular degeneration: a preliminary report.* Lancet, 2012. **379**(9817): p. 713-20.
6. Hood, D.C., et al., *ISCEV standard for clinical multifocal electroretinography (mfERG) (2011 edition).* Doc Ophthalmol, 2012. **124**(1): p. 1-13.
7. Odom, J.V., et al., *ISCEV standard for clinical visual evoked potentials: (2016 update).* Doc Ophthalmol, 2016. **133**(1): p. 1-9.
8. Serino, F., et al., *Role of Vitreous Detachment in Epiretinal Membrane Peeling: A Multimodal Imaging and Microperimetry Study.* J Clin Med, 2024. **13**(12).
